# Supplementary material for: Long-term risk of arrhythmias in patients with inflammatory bowel disease: A population-based, sibling-controlled cohort study
Source: PLoS Med. 2023 Oct 19;20(10):e1004305. doi: 10.1371/journal.pmed.1004305 (PMC10621936; doi:10.1371/journal.pmed.1004305)
Supplement: S1 Appendix — Figure A. Hazard ratio (HR) and 95% confidence interval (CI) of specific arrhythmias, comparing inflammatory bowel disease patients with their reference individuals. Figure B. Standardized cumulative incidence and 95% CI of specific arrhythmias in inflammatory bowel disease patients (pink) and their reference individuals (blue). Table A. Previous important studies of inflammatory bowel disease and arrhythmias. Table B. International Classification of Disease (ICD) codes and SNOMED codes defining inflammatory bowel disease. Table C. ICD codes assigned for phenotypes of inflammatory bowel disease. Table D. Definitions of primary and secondary outcomes according to ICD codes. Table E. Definitions of comorbidities according to ICD codes. Table F. Definitions of prescription medications according to ATC codes. Table G. Cumulative incidence difference (95% CI) of arrhythmias during follow-up in individuals with inflammatory bowel disease, compared with their matched reference individuals. Table H. Incident overall arrhythmias in patients with inflammatory bowel disease and their matched reference individuals, stratified by sex, age at index date, calendar period, educational attainment, and number of healthcare visits. Table I. Incident overall arrhythmias in patients with inflammatory bowel disease and their matched reference individuals, stratified by the phenotypes of the Montreal Classification. Table J. Incident specific arrhythmias in patients with inflammatory bowel disease and their matched reference individuals, stratified by the phenotypes of the Montreal Classification. Table K. Sensitivity analyses of the incident arrhythmia in patients with inflammatory bowel disease and their matched reference individuals. Table L. Incident arrhythmia in patients with inflammatory bowel disease and their matched reference individuals (1-year or 3-years lag time). Table M. Characteristics of patients with inflammatory bowel disease and their IBD-free full siblings. Table N. I [file pmed.1004305.s002.docx]

**Long-term risk of arrhythmias in patients with inflammatory bowel disease: A population-based, sibling-controlled cohort study**

J Sun et al.

**Content**

Figure A: Hazard ratio (HR) and 95% confidence interval (CI) of specific arrhythmias, comparing inflammatory bowel disease patients with their reference individuals.

Figure B: Standardized cumulative incidence and 95% confidence interval of specific arrhythmias in inflammatory bowel disease patients (pink) and their reference individuals (blue).

Table A. Previous important studies of inflammatory bowel disease and arrhythmias.

Table B. International Classification of Disease (ICD) codes and SNOMED codes defining inflammatory bowel disease.

Table C. ICD codes assigned for phenotypes of inflammatory bowel disease.

Table D. Definitions of primary and secondary outcomes according to ICD codes.

Table E. Definitions of comorbidities according to ICD codes.

Table F. Definitions of prescription medications according to ATC codes.

Table G. Cumulative incidence difference (95%CI) of arrhythmias during follow-up in individuals with inflammatory bowel disease, compared with their matched reference individuals.

Table H. Incident overall arrhythmias in patients with inflammatory bowel disease and their matched reference individuals, stratified by sex, age at index date, calendar period, educational attainment, and number of healthcare visits.

Table I. Incident overall arrhythmias in patients with inflammatory bowel disease and their matched reference individuals, stratified by the phenotypes of the Montreal Classification.

Table J. Incident specific arrhythmias in patients with inflammatory bowel disease and their matched reference individuals, stratified by the phenotypes of the Montreal Classification.

Table K. Sensitivity analyses of the incident arrhythmia in patients with inflammatory bowel disease and their matched reference individuals.

Table L. Incident arrhythmia in patients with inflammatory bowel disease and their matched reference individuals (1-year or 3-years lag time).

Table M. Characteristics of patients with inflammatory bowel disease and their IBD-free full siblings.

Table N. Incident arrhythmia in patients with inflammatory bowel disease and their IBD-free full siblings.


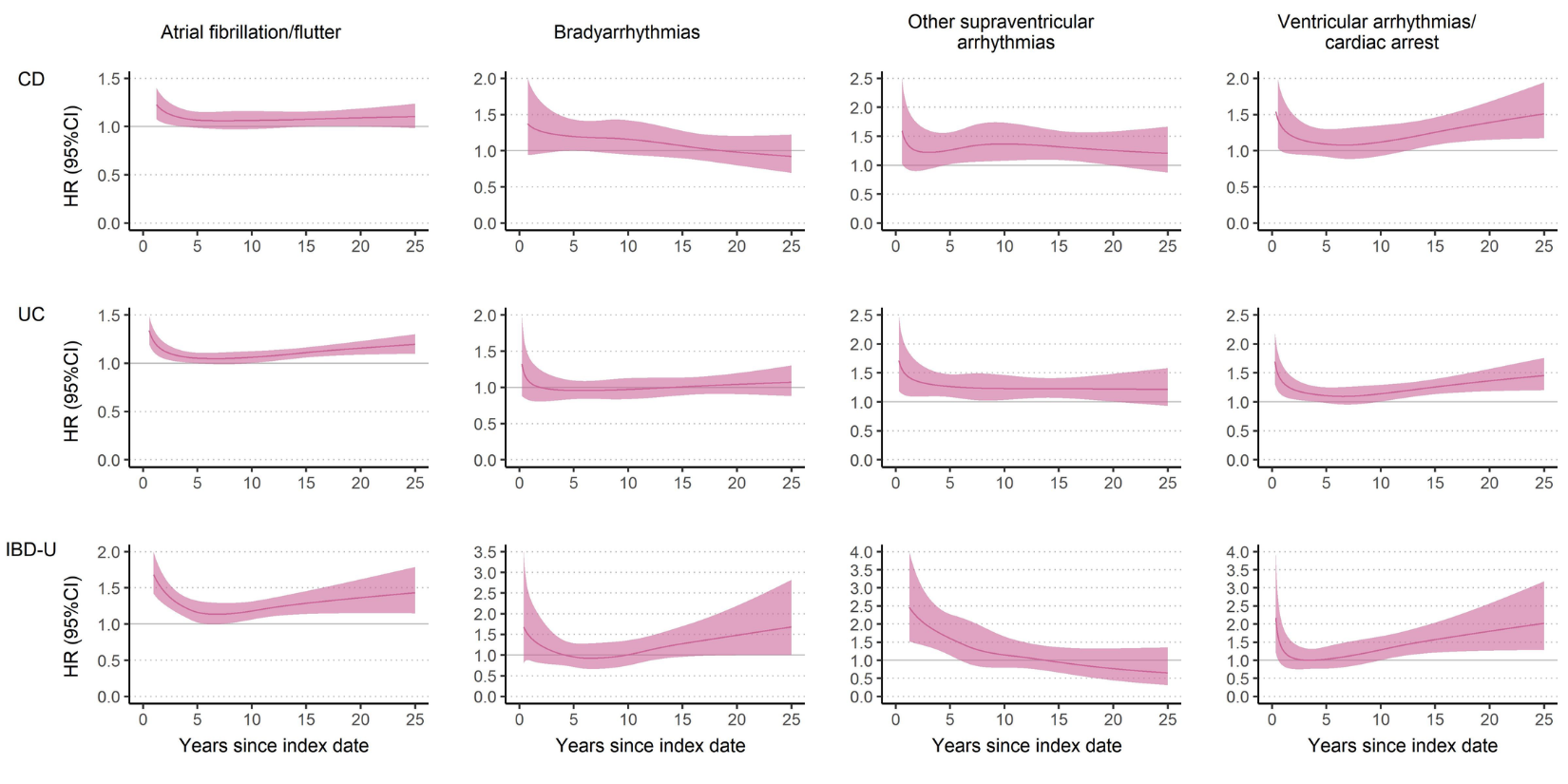


Figure A: Hazard ratio (HR) and 95% confidence interval (CI) of specific arrhythmias, comparing inflammatory bowel disease patients with their reference individuals. CD: Crohn's disease; IBD-U: inflammatory bowel disease unclassified; UC: ulcerative colitis.


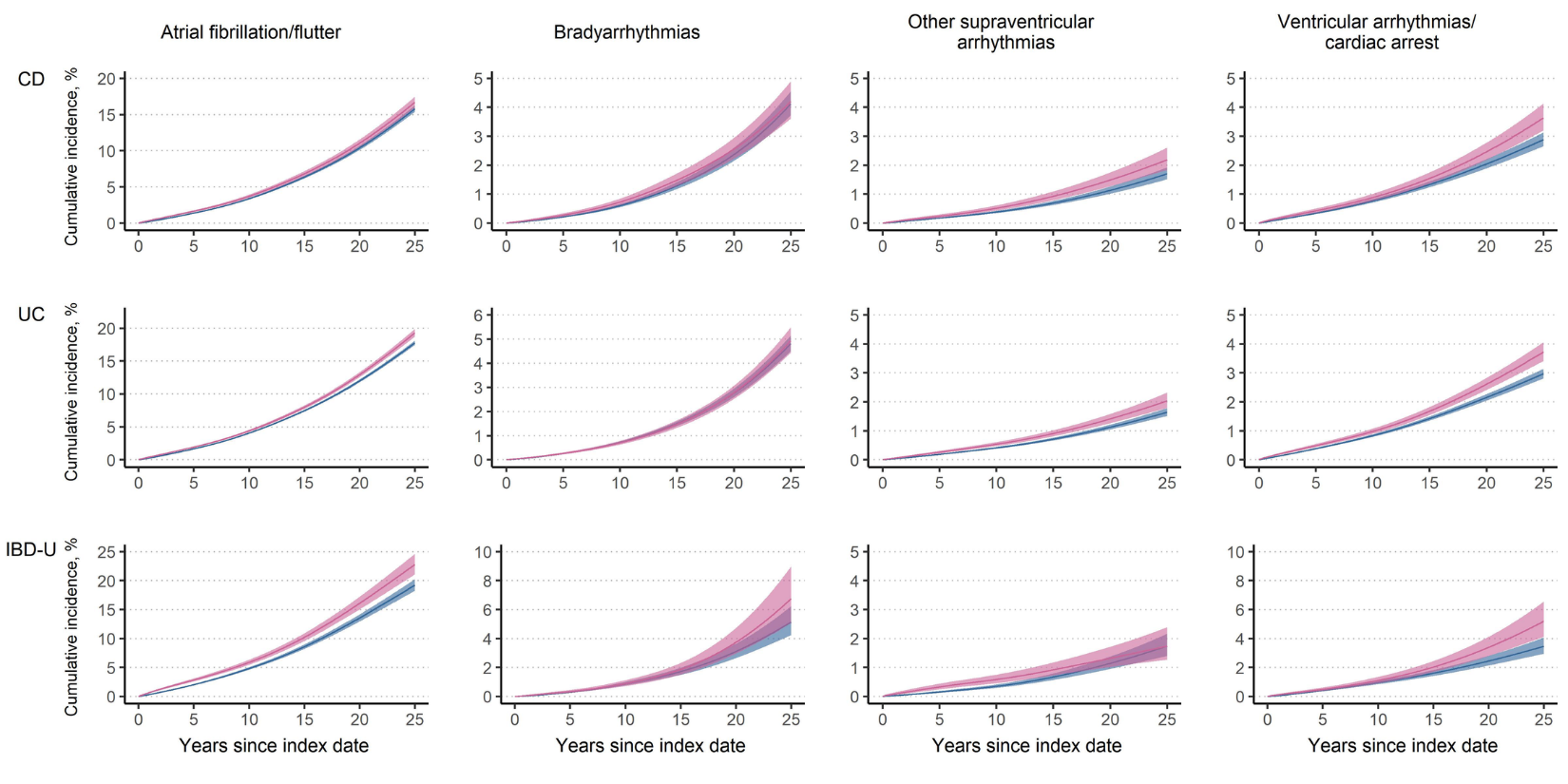


Figure B: Standardized cumulative incidence and 95% confidence interval of specific arrhythmias in inflammatory bowel disease patients (pink) and their reference individuals (blue). CD: Crohn's disease; IBD-U: inflammatory bowel disease unclassified; UC: ulcerative colitis.

| **Table A. Previous important studies of inflammatory bowel disease and arrhythmias** | | | | | | | |
| --- | --- | --- | --- | --- | --- | --- | --- |
| Study type, first author, publication year, country | Study period | Sample size | Male, % | Mean age, years | Follow-up time, years | No. of events | Main findings |
| Cross-sectional, Mubasher, 2020, USA[1] | 2012-2014 | IBD:169,447; general population: 16,951,470 | - | IBD: 52.4; general population: 57.4 | - | IBD: 9.7%; general population: 14.2% | Lower rates of hospitalization-related-arrhythmias in the IBD population, OR=0.87 (95% CI: 0.85, 0.88) |
| Population-based cohort, Choi, 2019, Korea[2] | 2010-2014 | IBD:37,696 (CD: 12,349; UC: 25,397); age- and sex-matched controls: 113,088 | 61.0 | IBD and controls: 39.4 | 4.9 ± 1.3 | 1,120 (IBD: 348; control: 772) | Higher risk of AF in patients with IBD, HR=1.36 (95% CI: 1.20, 1.54); much higher in those with CD, younger patients, those without CVD risk factors, and those with moderate-to-severe IBD (i.e., those with IBD medication use) |
| Review of the clinical records, Pattanshetty, 2015, USA[3] | 2001-2010 | IBD: 142 | 36.6 | IBD: 56.4 | - | IBD: 16 (11.3%) vs 0.9% in the USA general population | Higher AF prevalence in patients with IBD compared to those in a large cross-sectional study (n=1.89 million) to evaluate AF prevalence in USA population |
| Nationwide cohort, Kristensen, 2014, Demark[4] | 1996-2011 | IBD: 24,499 (CD: 6,668; UC: 17,831); age- and sex-matched control: 236,275 | 46.1 | IBD and control: 43.9 | 6.8 | IBD: 685; control: 4,390; incident rate: 4.16 vs 2.70 per 1000 person-years | Overall IBD-associated risk of AF, IRR=1.26 (95% CI: 1.16, 1.36), mainly driven by increased AF incidence during IBD flares [IRR=2.63 (95% CI: 2.26, 3.06)] and persistent activity [IRR=2.06 (95% CI: 1.67, 2.55)], no increased AF risk in remission periods [IRR=0.97 (95% CI: 0.88, 1.08)]. |

AF: atrial fibrillation; CD: Crohn's disease; CI: confidence interval; CVD: cardiovascular disease; HR: hazard ratio; IBD: inflammatory bowel disease; IRR: incidence rate ratio; OR: odds ratio; UC: ulcerative colitis, USA: the United States of America.

| **Table B. International Classification of Disease (ICD) codes and SNOMED codes defining inflammatory bowel disease (IBD) ^a^**. | | | | | |
| --- | --- | --- | --- | --- | --- |
|  | ICD-7 (1964-1968) | ICD-8 (1969-1986) | ICD-9 (1987-1996) | ICD-10 (1997-) | SNOMED codes ^b^ |
| Ulcerative colitis (UC) | 572,20; 572,21; 578,03 | 563,1; 563,10; 569,02; 569,04 | 556 | K51 | D6255 or M41, M42, M43, M44, M463, or M47 |
| Crohn’s disease (CD) | 572,00; 572,09 | 563,00 | 555 | K50 | D6216 or M41, M42, M43, M44, M463, or M47 |
| IBD unclassified (IBD-U) | UC + CD | UC + CD or 563; 563,0; 563,9; 563,98; 563,99 | UC + CD | K52.3 | D6214 or M41, M42, M43, M44, M463, or M47 |
| SNOMED: Systematized Nomenclature of Medicine. | | | | | |
| ^a^ ≥1 ICD code for IBD plus a relevant biopsy code has a positive predictive value of 95%[5,6]. | | | | | |
| ^b^ D codes are diagnostic codes but listed under morphology in pathology registers; D6255 for example is the diagnostic code for UC. SNOMED codes starting with "M"; M41 for example refers to all codes starting with "M41". | | | | | |
| Because definitions of exposure should not “look into the future”, IBD subtypes were defined in accordance with the first two diagnostic codes only (i.e., no information after start of follow-up contributed to the subtype definition). For individuals with one ICD code for IBD and one unspecific SNOMED code (e.g., “M…”), the IBD subtype was determined by the ICD code in the patient register only. In a recent paper[7], we report that 18% of incident patients with IBD in the Swedish patient register during 2002-2014 were classified as another IBD subtype at some point during follow-up. | | | | | |

| **Table C. ICD codes assigned for phenotypes of inflammatory bowel disease** | |
| --- | --- |
| Montreal classification | Diagnostic codes |
| Crohn’s disease location ^a^ |  |
| Ileal (L1) | K50.0 |
| Colonic (L2) | K50.1 |
| Ileocolonic or location not defined (L3/LX) | K50.8, K50.9 |
| Ulcerative colitis extent ^a^ |  |
| Proctitis (E1) | K51.2 |
| Left-sided colitis (E2) | K51.3; K51.5 |
| Extensive colitis (E3) | K51.0 |
| Extent not defined (EX) | K51.4; K51.8; K51.9 |
| Primary sclerosing cholangitis (PSC) ^b^ | ICD-9 (1987-1996): 576B |
|  | ICD-10 (1997-): K830 |
| Other extraintestinal manifestations | ICD-9: 695C, 364, 713B, 720A, 720C, 720W, 720X |
|  | ICD-10: L52, L88, L98.2, H20, M07.4, M07.5, M07.6, M09.1, M09.2, M45, M460, M461, M468, M469, M139, M255 |
| E: Extent; ICD: International Classification of Diseases; L: location. | |
| ^a^ Validated definitions and diagnostic codes[8] used to define Crohn’s disease and ulcerative colitis according to the Montreal classification since the start of use of the ICD-10 in Sweden (1997-). All codes are captured in the Swedish National Patient Register (prospectively recorded in routine clinical practice). | |
| ^b^ We restricted our use to ICD-9 and ICD-10 codes since we believe that earlier ICD codes for extraintestinal inflammation are less reliable, particularly for primary sclerosing cholangitis. The validity of the PSC codes has not formally been tested in Sweden[9]. | |

| **Table D. Definitions of primary and secondary outcomes according to ICD codes** | |
| --- | --- |
| Outcome | Definition |
| Primary outcome |  |
| Overall arrhythmia | Composite outcome including any individual outcomes from 1a-1d. |
| Secondary outcomes |  |
| 1a. Atrial fibrillation/flutter | ICD-8: 427,92 |
|  | ICD-9: 427D |
|  | ICD-10: I48 |
| 1b. Bradyarrhythmias | ICD-8: 427,20; 427,27; 427,28; 427,29 |
|  | ICD-9: 426A; 426B; 426G; 426X |
|  | ICD-10: I441; I442; I452; I453; I459; I495 |
| 1c. Other supraventricular arrhythmias | ICD-8: 427,90 |
|  | ICD-9: 426H; 427A |
|  | ICD-10: I456; I471 |
| 1d. Ventricular arrhythmias/cardiac arrest | ICD-8: 427,91; 795,99 |
|  | ICD-9: 427B; 427E; 427F; 798B; 798C |
|  | ICD-10: I460; I461; I469; I470; I472; I490; R960 |

ICD: International Classification of Diseases.

| **Table E. Definitions of comorbidities according to ICD codes** | |
| --- | --- |
| **Comorbidity** | **Definition** |
| Ischemic heart disease | ICD-8: 410-414 |
|  | ICD-9: 410-414 |
|  | ICD-10: I20-25 |
| Heart failure | ICD-8: 428 |
|  | ICD-9: 428 |
|  | ICD-10: I42; I50 |
| Stroke | ICD-8: 431-434 |
|  | ICD-9: 431-434 |
|  | ICD-10: I61-64 |
| Hypertension | ICD-8: 400-404 |
|  | ICD-9: 401-405 |
|  | ICD-10: I10-I15 |
|  | Medication ATC codes: see antihypertensive medications in Table F |
| Obesity | ICD-8: 277 |
|  | ICD-9: 278A, 278B |
|  | ICD-10: E65-66 |
| Diabetes | ICD-8: 250 |
|  | ICD-9: 250 |
|  | ICD-10: E10-E14, O24 |
|  | Medication ATC codes: see antidiabetic medications in Table F |
| Dyslipidemia | ICD-8: 279 |
|  | ICD-9: 272 |
|  | ICD-10: E78 |
|  | Medication ATC codes: C10 |
| Chronic kidney disease | ICD-8: 585, 586, Y29,01 |
|  | ICD-9: 585, 586, 753B, V42A, V45B, V56 |
|  | ICD-10: N18, N19; N26; T824; Y841; Q61; Z49; Z992; Z940 |
| Chronic obstructive pulmonary disease (COPD)  (only if patient diagnosed ≥40 years old) | ICD-8: 491, 492 |
|  | ICD-9: 491, 492, 496 |
|  | ICD-10: J41-J44 |
| Autoimmune thyroid disease | ICD-8: 242,0; 245,02; 245,03 |
|  | ICD-9: 242A, 245C |
|  | ICD-10: E050, E063 |

ATC: Anatomical Therapeutic Chemical; ICD: International Classification of Diseases.

| **Table F. Definitions of prescription medications according to ATC codes** | |
| --- | --- |
| **Covariates** | **Definition** |
| Aspirin | B01AC06 |
| Non-aspirin anti-platelet medications | B01AC excluding aspirin (B01AC06) |
| Statins | C10AA |
| Non-statin lipid lowering medications | C10AB, C10AC, C10AD, C10AX01-14 |
| Anticoagulation medications | B01AA, B01AE, B01AF, B01AX |
| Antidiabetic medications | A10 |
| Antihypertensive medications | C02, C03AA-AB, C03BA, C03CA, C03DA, C03EA, C08CA, C08DA, C08DB, C09A, C09BA, C09BB, C09CA, C09DA, C09DB01 |

ATC: Anatomical Therapeutic Chemical.

| **Table G. Cumulative incidence difference (95%CI) of arrhythmias during follow-up in individuals with inflammatory bowel disease, compared with their matched reference individuals** | | | | |
| --- | --- | --- | --- | --- |
| Outcomes | Years since the index date | | | |
|  | 1 year | 5 years | 10 years | 25 years |
| CD | | | | |
| Overall arrhythmias | 0.19 (0.12, 0.26) | 0.35 (0.18, 0.51) | 0.48 (0.22, 0.74) | 1.25 (0.48, 2.01) |
| Atrial fibrillation/flutter | 0.14 (0.08, 0.20) | 0.26 (0.11, 0.40) | 0.34 (0.10, 0.57) | 0.89 (0.15, 1.63) |
| Bradyarrhythmias | 0.02 (-0.00, 0.04) | 0.06 (-0.00, 0.12) | 0.12 (0.01, 0.24) | 0.08 (-0.53, 0.69) |
| Other supraventricular arrhythmias | 0.02 (0.00, 0.05) | 0.06 (0.00, 0.12) | 0.13 (0.04, 0.23) | 0.48 (0.12, 0.85) |
| Ventricular arrhythmias/cardiac arrest | 0.04 (0.01, 0.08) | 0.08 (0.00, 0.16) | 0.12 (-0.01, 0.24) | 0.76 (0.31, 1.21) |
| UC | | | | |
| Overall arrhythmias | 0.21 (0.15, 0.26) | 0.39 (0.26, 0.52) | 0.48 (0.28, 0.68) | 1.73 (1.12, 2.33) |
| Atrial fibrillation/flutter | 0.15 (0.10, 0.20) | 0.26 (0.15, 0.37) | 0.34 (0.15, 0.52) | 1.52 (0.93, 2.11) |
| Bradyarrhythmias | 0.01 (-0.01, 0.02) | 0.00 (-0.04, 0.05) | -0.01 (-0.10, 0.07) | 0.12 (-0.38, 0.63) |
| Other supraventricular arrhythmias | 0.02 (0.01, 0.04) | 0.07 (0.03, 0.12) | 0.13 (0.05, 0.20) | 0.40 (0.13, 0.66) |
| Ventricular arrhythmias/cardiac arrest | 0.05 (0.02, 0.08) | 0.10 (0.04, 0.16) | 0.15 (0.05, 0.24) | 0.76 (0.43, 1.08) |
| IBD-U | | | | |
| Overall arrhythmias | 0.44 (0.31, 0.58) | 0.99 (0.69, 1.29) | 1.24 (0.74, 1.75) | 3.48 (1.72, 5.23) |
| Atrial fibrillation/flutter | 0.34 (0.22, 0.46) | 0.81 (0.54, 1.09) | 1.06 (0.59, 1.53) | 3.57 (1.80, 5.33) |
| Bradyarrhythmias | 0.02 (-0.01, 0.05) | 0.05 (-0.06, 0.15) | 0.02 (-0.20, 0.24) | 1.62 (-0.20, 3.44) |
| Other supraventricular arrhythmias | 0.05 (0.01, 0.10) | 0.17 (0.07, 0.28) | 0.24 (0.08, 0.39) | -0.01 (-0.58, 0.57) |
| Ventricular arrhythmias/cardiac arrest | 0.07 (0.01, 0.12) | 0.08 (-0.05, 0.20) | 0.15 (-0.08, 0.39) | 1.74 (0.60, 2.89) |
| CD: Crohn's disease; CI: confidence interval; IBD-U: inflammatory bowel disease unclassified; UC: ulcerative colitis. | | | | |
| Cumulative incidence difference was estimated from the flexible parametric model, allowing the effect of IBD to vary over time. All models were conditioned on the matching variables (birth year, sex, county of residence, and calendar year) and further adjusted for country of birth, educational attainment, number of healthcare visits, ischemic heart disease, heart failure, stroke, hypertension, diabetes, obesity, dyslipidemia, chronic kidney disease, and chronic obstructive pulmonary disease . | | | | |

| **Table H. Incident overall arrhythmias in patients with inflammatory bowel disease and their matched population references, stratified by sex, age at index date, calendar period, educational attainment, and number of healthcare visits** | | | | | | | |
| --- | --- | --- | --- | --- | --- | --- | --- |
|  | No. of events, n (%) | | IR (95%CI), per 10,000 Pys | | IR (95%CI) difference, per 10,000 Pys | HR (95%CI) | |
|  | Patients | References | Patients | References |  | Model 1 ^a^ | Model 2 ^b^ |
| CD | | | | | | | |
| Sex |  |  |  |  |  |  |  |
| Male | 1008 (8.5) | 4320 (7.6) | 61.6 (57.9, 65.5) | 53.2 (51.6, 54.8) | 8.4 (4.3, 12.5) | 1.18 (1.10, 1.27) | 1.10 (1.02, 1.19) |
| Female | 896 (6.9) | 3713 (5.9) | 48.4 (45.3, 51.6) | 40.0 (38.7, 41.3) | 8.4 (5.0, 11.8) | 1.34 (1.24, 1.45) | 1.20 (1.11, 1.31) |
| Age at index date, years |  |  |  |  |  |  |  |
| <18 | 23 (0.8) | 95 (0.7) | 5.5 (3.5, 8.3) | 4.6 (3.7, 5.6) | 1.0 (-1.5, 3.4) | 1.26 (0.79, 1.99) | 1.27 (0.79, 2.06) |
| 18-39 | 260 (2.5) | 978 (1.9) | 15.6 (13.8, 17.7) | 11.9 (11.2, 12.7) | 3.7 (1.7, 5.8) | 1.34 (1.16, 1.54) | 1.20 (1.04, 1.39) |
| 40-59 | 681 (9.5) | 3045 (8.8) | 66.8 (61.9, 72.0) | 59.0 (56.9, 61.1) | 7.8 (2.4, 13.3) | 1.19 (1.10, 1.30) | 1.09 (0.99, 1.19) |
| ≥60 | 940 (19.9) | 3915 (19.2) | 241.0 (226.0, 256.7) | 200.6 (194.4, 206.9) | 40.4 (23.9, 56.8) | 1.27 (1.18, 1.38) | 1.17 (1.08, 1.27) |
| Calendar period at index date |  |  |  |  |  |  |  |
| 1969-1989 | 249 (11.7) | 1340 (12.7) | 41.7 (36.7, 47.2) | 44.0 (41.7, 46.4) | -2.3 (-8.0, 3.4) | 1.04 (0.90, 1.21) | 1.00 (0.86, 1.17) |
| 1990-1999 | 646 (11.3) | 2828 (10.2) | 56.3 (52.0, 60.8) | 48.5 (46.7, 50.3) | 7.8 (3.2, 12.5) | 1.30 (1.19, 1.43) | 1.22 (1.11, 1.34) |
| 2000-2009 | 771 (7.9) | 3027 (6.5) | 59.1 (55.0, 63.4) | 47.2 (45.5, 48.9) | 11.9 (7.5, 16.4) | 1.28 (1.17, 1.39) | 1.15 (1.05, 1.26) |
| 2010-2019 | 238 (3.2) | 838 (2.4) | 54.1 (47.5, 61.5) | 39.6 (37.0, 42.4) | 14.5 (7.2, 21.9) | 1.30 (1.12, 1.51) | 1.14 (0.97, 1.34) |
| Educational attainment, years |  |  |  |  |  |  |  |
| 0-9 | 625 (11.0) | 2575 (10.2) | 87.2 (80.5, 94.3) | 76.4 (73.5, 79.4) | 10.8 (3.3, 18.2) | 1.23 (1.08, 1.38) | 1.15 (1.01, 1.31) |
| 10-12 | 680 (7.1) | 2603 (6.0) | 53.0 (49.1, 57.1) | 43.3 (41.6, 45.0) | 9.7 (5.4, 14.0) | 1.31 (1.18, 1.47) | 1.20 (1.06, 1.34) |
| ≥13 | 297 (5.9) | 1346 (4.8) | 46.8 (41.7, 52.5) | 37.0 (35.1, 39.1) | 9.8 (4.1, 15.5) | 1.36 (1.13, 1.65) | 1.22 (0.99, 1.49) |
| Missing | 302 (6.4) | 1509 (6.6) | 35.4 (31.5, 39.6) | 34.3 (32.6, 36.1) | 1.1 (-3.3, 5.4) | 1.06 (0.92, 1.22) | 1.01 (0.88, 1.17) |
| Number of healthcare visits |  |  |  |  |  |  |  |
| 0 | 1081 (7.7) | 6163 (6.8) | 47.5 (44.7, 50.4) | 43.0 (41.9, 44.1) | 4.5 (1.4, 7.5) | 1.16 (1.08, 1.25) | 1.12 (1.04, 1.21) |
| 1 | 299 (7.3) | 916 (6.3) | 55.3 (49.2, 61.9) | 52.8 (49.5, 56.3) | 2.5 (-4.6, 9.6) | 0.96 (0.71, 1.29) | 0.93 (0.68, 1.27) |
| 2-3 | 260 (7.6) | 572 (6.4) | 69.4 (61.3, 78.4) | 66.0 (60.7, 71.6) | 3.4 (-6.6, 13.4) | 0.89 (0.65, 1.22) | 0.82 (0.58, 1.16) |
| ≥4 | 264 (7.9) | 382 (6.7) | 89.3 (78.9, 100.7) | 78.8 (71.1, 87.0) | 10.6 (-2.7, 23.9) | 1.77 (1.27, 2.48) | 1.94 (1.33, 2.82) |
| UC | | | | | | | |
| Sex |  |  |  |  |  |  |  |
| Male | 2534 (10.1) | 10349 (8.7) | 73.9 (71.1, 76.8) | 61.7 (60.5, 62.9) | 12.2 (9.1, 15.3) | 1.17 (1.12, 1.23) | 1.11 (1.06, 1.17) |
| Female | 1620 (7.5) | 6428 (6.2) | 54.2 (51.6, 56.9) | 43.8 (42.7, 44.9) | 10.4 (7.6, 13.3) | 1.27 (1.20, 1.35) | 1.19 (1.12, 1.26) |
| Age at index date, years |  |  |  |  |  |  |  |
| <18 | 25 (0.8) | 127 (0.8) | 5.2 (3.3, 7.6) | 5.3 (4.4, 6.3) | -0.1 (-2.3, 2.1) | 0.99 (0.64, 1.53) | 0.86 (0.53, 1.37) |
| 18-39 | 473 (2.5) | 1754 (1.9) | 16.6 (15.1, 18.1) | 12.4 (11.9, 13.0) | 4.1 (2.5, 5.8) | 1.38 (1.24, 1.53) | 1.31 (1.17, 1.45) |
| 40-59 | 1433 (9.5) | 6088 (8.4) | 65.4 (62.1, 68.9) | 56.7 (55.3, 58.1) | 8.8 (5.1, 12.4) | 1.17 (1.10, 1.24) | 1.10 (1.04, 1.17) |
| ≥60 | 2223 (21.8) | 8808 (20.2) | 250.2 (240.1, 260.7) | 209.8 (205.5, 214.2) | 40.4 (29.2, 51.5) | 1.20 (1.14, 1.27) | 1.13 (1.07, 1.19) |
| Calendar period at index date |  |  |  |  |  |  |  |
| 1969-1989 | 523 (16.9) | 2222 (14.6) | 65.1 (59.6, 70.9) | 54.4 (52.2, 56.7) | 10.7 (4.7, 16.7) | 1.30 (1.17, 1.44) | 1.24 (1.11, 1.38) |
| 1990-1999 | 1371 (12.8) | 5981 (11.6) | 65.2 (61.8, 68.7) | 56.9 (55.5, 58.3) | 8.3 (4.5, 12.0) | 1.19 (1.12, 1.27) | 1.12 (1.05, 1.20) |
| 2000-2009 | 1743 (8.7) | 6896 (7.2) | 64.1 (61.2, 67.2) | 52.7 (51.4, 53.9) | 11.4 (8.2, 14.7) | 1.17 (1.11, 1.24) | 1.11 (1.05, 1.17) |
| 2010-2019 | 517 (4.0) | 1678 (2.7) | 65.5 (60.0, 71.4) | 44.5 (42.4, 46.7) | 21.0 (14.9, 27.0) | 1.30 (1.18, 1.45) | 1.17 (1.05, 1.30) |
| Educational attainment, years |  |  |  |  |  |  |  |
| 0-9 | 1422 (14.2) | 5690 (11.8) | 112.5 (106.8, 118.5) | 89.5 (87.2, 91.8) | 23.0 (16.8, 29.3) | 1.17 (1.08, 1.26) | 1.10 (1.02, 1.20) |
| 10-12 | 1384 (7.3) | 5492 (6.2) | 54.6 (51.7, 57.5) | 45.7 (44.5, 46.9) | 8.9 (5.8, 12.0) | 1.16 (1.07, 1.25) | 1.09 (1.00, 1.18) |
| ≥13 | 744 (6.3) | 2986 (5.3) | 49.7 (46.2, 53.4) | 41.1 (39.6, 42.6) | 8.6 (4.7, 12.4) | 1.19 (1.05, 1.35) | 1.12 (0.98, 1.27) |
| Missing | 604 (9.9) | 2609 (8.6) | 54.1 (49.8, 58.5) | 44.9 (43.2, 46.7) | 9.1 (4.5, 13.8) | 1.27 (1.15, 1.40) | 1.21 (1.09, 1.34) |
| Number of healthcare visits |  |  |  |  |  |  |  |
| 0 | 2555 (8.8) | 12852 (7.6) | 57.5 (55.3, 59.8) | 49.8 (49.0, 50.7) | 7.7 (5.3, 10.1) | 1.18 (1.13, 1.24) | 1.15 (1.10, 1.21) |
| 1 | 691 (9.0) | 1871 (7.0) | 70.3 (65.1, 75.7) | 59.4 (56.8, 62.2) | 10.8 (5.0, 16.7) | 1.22 (1.01, 1.46) | 1.24 (1.03, 1.51) |
| 2-3 | 487 (8.6) | 1209 (7.2) | 81.2 (74.2, 88.7) | 73.4 (69.3, 77.6) | 7.8 (-0.5, 16.1) | 1.13 (0.87, 1.45) | 1.14 (0.88, 1.49) |
| ≥4 | 421 (9.5) | 845 (8.4) | 107.4 (97.5, 118.1) | 97.2 (90.8, 103.9) | 10.3 (-1.9, 22.4) | 1.35 (1.04, 1.77) | 1.48 (1.11, 1.98) |
| IBD-U | | | | | | | |
| Sex |  |  |  |  |  |  |  |
| Male | 544 (9.0) | 1905 (6.7) | 85.5 (78.5, 92.9) | 60.5 (57.8, 63.3) | 25.0 (17.3, 32.6) | 1.44 (1.30, 1.60) | 1.28 (1.15, 1.43) |
| Female | 446 (7.4) | 1468 (5.2) | 70.6 (64.2, 77.4) | 46.5 (44.1, 48.9) | 24.1 (17.2, 31.1) | 1.49 (1.32, 1.67) | 1.33 (1.17, 1.50) |
| Age at index date, years |  |  |  |  |  |  |  |
| <18 | 8 (0.7) | 22 (0.4) | 5.7 (2.5, 11.3) | 3.2 (2.0, 4.8) | 2.6 (-1.6, 6.7) | 1.82 (0.80, 4.13) | 1.92 (0.75, 4.94) |
| 18-39 | 86 (2.1) | 238 (1.2) | 17.1 (13.7, 21.2) | 9.6 (8.4, 10.9) | 7.5 (3.7, 11.4) | 1.75 (1.36, 2.25) | 1.49 (1.13, 1.95) |
| 40-59 | 309 (8.7) | 1080 (6.3) | 74.4 (66.4, 83.1) | 51.3 (48.3, 54.4) | 23.1 (14.3, 31.9) | 1.51 (1.32, 1.72) | 1.35 (1.17, 1.55) |
| ≥60 | 587 (18.5) | 2033 (15.4) | 277.6 (255.8, 300.6) | 197.8 (189.4, 206.5) | 79.8 (56.0, 103.5) | 1.39 (1.25, 1.54) | 1.22 (1.10, 1.37) |
| Calendar period at index date |  |  |  |  |  |  |  |
| 1969-1989 | 58 (17.4) | 196 (12.0) | 66.7 (50.7, 86.2) | 44.0 (38.1, 50.6) | 22.7 (4.5, 40.9) | 1.96 (1.42, 2.70) | 1.79 (1.26, 2.54) |
| 1990-1999 | 216 (13.2) | 833 (10.6) | 70.5 (61.5, 80.5) | 52.5 (49.0, 56.2) | 18.0 (8.0, 28.0) | 1.43 (1.21, 1.68) | 1.35 (1.13, 1.60) |
| 2000-2009 | 427 (9.3) | 1570 (7.3) | 75.2 (68.2, 82.6) | 56.3 (53.5, 59.1) | 18.9 (11.3, 26.5) | 1.30 (1.16, 1.46) | 1.17 (1.03, 1.32) |
| 2010-2019 | 289 (5.2) | 774 (3.0) | 94.2 (83.7, 105.6) | 52.1 (48.5, 55.8) | 42.1 (30.7, 53.5) | 1.70 (1.47, 1.97) | 1.44 (1.23, 1.69) |
| Educational attainment, years |  |  |  |  |  |  |  |
| 0-9 | 335 (12.4) | 1206 (9.8) | 120.7 (108.2, 134.3) | 89.4 (84.4, 94.5) | 31.3 (17.6, 45.1) | 1.38 (1.16, 1.65) | 1.19 (0.98, 1.43) |
| 10-12 | 365 (7.7) | 1218 (5.7) | 72.1 (64.9, 79.9) | 50.7 (47.9, 53.6) | 21.4 (13.5, 29.3) | 1.39 (1.18, 1.63) | 1.25 (1.06, 1.48) |
| ≥13 | 193 (6.6) | 694 (4.6) | 64.7 (55.9, 74.4) | 43.6 (40.4, 46.9) | 21.1 (11.5, 30.8) | 1.73 (1.34, 2.22) | 1.78 (1.35, 2.34) |
| Missing | 97 (5.8) | 255 (3.3) | 52.1 (42.3, 63.5) | 26.5 (23.3, 29.9) | 25.6 (14.8, 36.5) | 2.09 (1.56, 2.80) | 1.84 (1.34, 2.54) |
| Number of healthcare visits |  |  |  |  |  |  |  |
| 0 | 455 (7.6) | 2283 (5.8) | 62.8 (57.2, 68.8) | 47.2 (45.3, 49.2) | 15.6 (9.6, 21.7) | 1.38 (1.23, 1.56) | 1.35 (1.19, 1.52) |
| 1 | 173 (8.5) | 434 (5.6) | 81.9 (70.2, 95.0) | 59.7 (54.3, 65.6) | 22.2 (8.8, 35.6) | 0.95 (0.66, 1.36) | 0.91 (0.61, 1.34) |
| 2-3 | 162 (8.4) | 332 (6.0) | 92.1 (78.5, 107.3) | 72.4 (64.8, 80.6) | 19.7 (3.6, 35.8) | 1.24 (0.82, 1.87) | 1.31 (0.83, 2.07) |
| ≥4 | 200 (9.5) | 324 (8.4) | 127.6 (110.6, 146.5) | 113.3 (101.4, 126.3) | 14.3 (-7.1, 35.8) | 1.26 (0.88, 1.80) | 1.43 (0.93, 2.18) |
| CD: Crohn's disease; CI: confidence interval; HR: hazard ratio; IBD-U: inflammatory bowel disease unclassified; IR: incidence rate; UC: ulcerative colitis; Pys: person-years. | | | | | | | |
| ^a^ Conditioned on the matching variables (birth year, sex, county of residence, and calendar period). | | | | | | | |
| ^b^ Further adjusted for country of birth, educational attainment, number of healthcare visits, ischemic heart disease, heart failure, stroke, hypertension, diabetes, obesity, dyslipidemia, chronic kidney disease, and chronic obstructive pulmonary disease. | | | | | | | |

| **Table I. Incident overall arrhythmias in patients with inflammatory bowel disease and their matched reference individuals, stratified by the phenotypes of the Montreal Classification** | | | | | | | |
| --- | --- | --- | --- | --- | --- | --- | --- |
|  | No. of events, n (%) | | IR (95%CI), per 10,000 Pys | | IR (95%CI) difference, per 10,000 Pys | HR (95%CI) | |
|  | Patients | References | Patients | References |  | Model 1 ^a^ | Model 2 ^b^ |
| CD | | | | | | | |
| Montreal Classification CD |  |  |  |  |  |  |  |
| L1, L3/LX (Ileal, ileocolonic or location not defined) | 921 (6.1) | 3633 (5.1) | 57.3 (53.6, 61.1) | 45.8 (44.4, 47.4) | 11.4 (7.4, 15.4) | 1.28 (1.18, 1.38) | 1.15 (1.06, 1.25) |
| L2 (Colonic) | 240 (6.6) | 908 (5.2) | 56.6 (49.7, 64.2) | 43.5 (40.7, 46.4) | 13.1 (5.4, 20.7) | 1.32 (1.13, 1.54) | 1.20 (1.02, 1.41) |
| Perianal | 59 (4.5) | 210 (3.3) | 43.7 (33.3, 56.3) | 31.4 (27.3, 35.9) | 12.3 (0.4, 24.2) | 1.48 (1.09, 2.00) | 1.28 (0.91, 1.82) |
| Extraintestinal manifestations |  |  |  |  |  |  |  |
| Primary sclerosing cholangitis | 6 (3.9) | 52 (7.2) | 36.7 (13.5, 79.8) | 58.3 (43.6, 76.4) | -21.6 (-54.9, 11.7) | 0.80 (0.33, 1.97) | 0.39 (0.09, 1.62) |
| Other extraintestinal manifestations | 94 (6.5) | 327 (4.8) | 70.3 (56.8, 85.9) | 49.3 (44.1, 54.9) | 21.0 (5.9, 36.2) | 1.49 (1.17, 1.90) | 1.39 (1.06, 1.83) |
| UC | | | | | | | |
| Montreal Classification UC |  |  |  |  |  |  |  |
| E1/E2 (Proctitis, left-sided colitis) | 860 (6.4) | 3443 (5.4) | 60.1 (56.2, 64.2) | 50.4 (48.8, 52.1) | 9.7 (5.3, 14.0) | 1.10 (1.02, 1.19) | 1.05 (0.97, 1.14) |
| E3 (Extensive colitis) | 523 (7.2) | 1976 (5.7) | 60.5 (55.5, 65.9) | 47.0 (45.0, 49.1) | 13.5 (7.9, 19.1) | 1.27 (1.14, 1.40) | 1.20 (1.08, 1.33) |
| EX (Extent not defined) | 1245 (8.2) | 4787 (6.7) | 69.5 (65.7, 73.4) | 55.0 (53.4, 56.6) | 14.5 (10.4, 18.7) | 1.22 (1.14, 1.31) | 1.13 (1.05, 1.21) |
| Extraintestinal manifestations |  |  |  |  |  |  |  |
| Primary sclerosing cholangitis | 58 (7.9) | 232 (6.6) | 62.0 (47.1, 80.1) | 45.3 (39.7, 51.5) | 16.7 (-0.2, 33.7) | 1.51 (1.10, 2.06) | 1.34 (0.94, 1.91) |
| Other extraintestinal manifestations | 137 (7.8) | 465 (5.7) | 83.5 (70.1, 98.6) | 57.9 (52.8, 63.4) | 25.6 (10.7, 40.5) | 1.46 (1.19, 1.78) | 1.36 (1.08, 1.70) |
| IBD-U | | | | | | | |
| Extraintestinal manifestations |  |  |  |  |  |  |  |
| Primary sclerosing cholangitis | 9 (4.9) | 60 (6.9) | 48.0 (22.0, 91.0) | 56.9 (43.4, 73.2) | -8.9 (-43.3, 25.6) | 1.07 (0.51, 2.24) | 0.56 (0.20, 1.54) |
| Other extraintestinal manifestations | 62 (7.6) | 180 (4.8) | 97.0 (74.5, 124.2) | 56.0 (48.1, 64.7) | 41.0 (15.7, 66.4) | 2.07 (1.49, 2.87) | 1.89 (1.26, 2.83) |
| CD: Crohn's disease; CI: confidence interval; E: Extent; HR: hazard ratio; IBD-U: inflammatory bowel disease unclassified; IR: incidence rate; L: location; UC: ulcerative colitis; Pys: person-years. | | | | | | | |
| ^a^ Conditioned on the matching variables (birth year, sex, county of residence, and calendar year). | | | | | | | |
| ^b^ Further adjusted for country of birth, educational attainment, number of healthcare visits, ischemic heart disease, heart failure, stroke, hypertension, diabetes, obesity, dyslipidemia, chronic kidney disease, and chronic obstructive pulmonary disease . | | | | | | | |

| **Table J. Incident specific arrhythmias in patients with inflammatory bowel disease and their matched reference individuals, stratified by the phenotypes of the Montreal Classification** | | | | | | | | | | | | | | | | |
| --- | --- | --- | --- | --- | --- | --- | --- | --- | --- | --- | --- | --- | --- | --- | --- | --- |
|  | Atrial fibrillation/flutter | | |  | Bradyarrhythmias | | |  | Other supraventricular arrhythmias | | |  | Ventricular arrhythmias/cardiac arrest | | | |
|  | Patients, n (%) | References, n (%) | HR (95%CI) ^a^ |  | Patients, n (%) | References, n (%) | HR (95%CI) ^a^ |  | Patients, n (%) | References, n (%) | HR (95%CI) ^a^ |  | Patients, n (%) | References, n (%) | HR (95%CI) ^a^ |  |
|  | CD | | | | | | | | | | | | | | | |
| Montreal Classification CD |  |  |  |  |  |  |  |  |  |  |  |  |  |  |  |  |
| L1, L3/LX (Ileal, ileocolonic or location not defined) | 678 (4.5) | 2811 (3.9) | 1.10 (1.01, 1.21) |  | 139 (0.9) | 457 (0.6) | 1.24 (1.00, 1.53) |  | 108 (0.7) | 368 (0.5) | 1.42 (1.13, 1.79) |  | 133 (0.9) | 466 (0.7) | 1.17 (0.94, 1.47) |  |
| L2 (Colonic) | 185 (5.1) | 687 (3.9) | 1.22 (1.02, 1.47) |  | 39 (1.1) | 129 (0.7) | 1.35 (0.88, 2.08) |  | 22 (0.6) | 86 (0.5) | 1.13 (0.68, 1.89) |  | 38 (1.0) | 127 (0.7) | 1.13 (0.73, 1.75) |  |
| Perianal | 35 (2.7) | 153 (2.4) | 1.17 (0.75, 1.82) |  | 6 (0.5) | 23 (0.4) | 0.70 (0.17, 2.99) |  | 14 (1.1) | 30 (0.5) | 4.52 (1.77, 11.53) |  | 11 (0.8) | 30 (0.5) | 1.12 (0.37, 3.45) |  |
| Extraintestinal manifestations |  |  |  |  |  |  |  |  |  |  |  |  |  |  |  |  |
| Primary sclerosing cholangitis | 2 (1.3) | 45 (6.2) | NA |  | 3 (1.9) | 6 (0.8) | NA |  | 1 (0.6) | 1 (0.1) | NA |  | 1 (0.6) | 9 (1.2) | NA |  |
| Other extraintestinal manifestations | 68 (4.7) | 239 (3.5) | 1.52 (1.10, 2.10) |  | 21 (1.4) | 41 (0.6) | 2.45 (1.22, 4.91) |  | 8 (0.6) | 41 (0.6) | 0.72 (0.28, 1.85) |  | 12 (0.8) | 48 (0.7) | 0.93 (0.37, 2.33) |  |
|  | UC | | | | | | | | | | | | | | | |
| Montreal Classification UC |  |  |  |  |  |  |  |  |  |  |  |  |  |  |  |  |
| E1/E2 (Proctitis, left, sided colitis) | 671 (5.0) | 2649 (4.2) | 1.04 (0.95, 1.15) |  | 103 (0.8) | 416 (0.7) | 1.01 (0.79, 1.27) |  | 95 (0.7) | 354 (0.6) | 1.27 (1.00, 1.61) |  | 92 (0.7) | 439 (0.7) | 0.83 (0.65, 1.07) |  |
| E3 (Extensive colitis) | 420 (5.8) | 1536 (4.4) | 1.24 (1.10, 1.40) |  | 56 (0.8) | 292 (0.8) | 0.85 (0.62, 1.16) |  | 39 (0.5) | 189 (0.5) | 0.84 (0.57, 1.22) |  | 71 (1.0) | 227 (0.7) | 1.36 (1.01, 1.83) |  |
| EX (Extent not defined) | 967 (6.4) | 3810 (5.3) | 1.10 (1.02, 1.19) |  | 155 (1.0) | 662 (0.9) | 1.04 (0.86, 1.26) |  | 112 (0.7) | 394 (0.5) | 1.33 (1.07, 1.67) |  | 195 (1.3) | 539 (0.7) | 1.47 (1.22, 1.77) |  |
| Extraintestinal manifestations |  |  |  |  |  |  |  |  |  |  |  |  |  |  |  |  |
| Primary sclerosing cholangitis | 36 (4.9) | 190 (5.4) | 0.99 (0.64, 1.52) |  | 10 (1.4) | 33 (0.9) | 1.71 (0.67, 4.38) |  | 7 (1.0) | 18 (0.5) | 4.99 (1.28, 19.48) |  | 12 (1.6) | 27 (0.8) | 1.46 (0.44, 4.92) |  |
| Other extraintestinal manifestations | 107 (6.1) | 378 (4.6) | 1.21 (0.94, 1.55) |  | 13 (0.7) | 61 (0.7) | 1.20 (0.55, 2.59) |  | 19 (1.1) | 37 (0.5) | 2.94 (1.44, 5.98) |  | 20 (1.1) | 57 (0.7) | 1.60 (0.83, 3.10) |  |
|  | IBD-U | | | | | | | | | | | | | | | |
| Extraintestinal manifestations |  |  |  |  |  |  |  |  |  |  |  |  |  |  |  |  |
| Primary sclerosing cholangitis | 7 (3.8) | 43 (5.0) | 0.45 (0.11, 1.81) |  | 0 (0.0) | 6 (0.7) | NA |  | 1 (0.5) | 12 (1.4) | NA |  | 2 (1.1) | 10 (1.2) | NA |  |
| Other extraintestinal manifestations | 53 (6.5) | 146 (3.9) | 2.22 (1.40, 3.52) |  | 10 (1.2) | 21 (0.6) | 1.02 (0.25, 4.20) |  | 4 (0.5) | 15 (0.4) | 1.80 (0.26, 12.72) |  | 5 (0.6) | 25 (0.7) | NA |  |
| CD: Crohn's disease; CI: confidence interval; E: Extent; HR: hazard ratio; IBD-U: inflammatory bowel disease unclassified; L: location; NA: not available; UC: ulcerative colitis. | | | | | | | | | | | | | | | | |
| ^a^ Conditioned on the matching variables (birth year, sex, county of residence, and calendar year) and further adjusted for country of birth, educational attainment, number of healthcare visits, ischemic heart disease, heart failure, stroke, hypertension, diabetes, obesity, dyslipidemia, chronic kidney disease, and chronic obstructive pulmonary disease. | | | | | | | | | | | | | | | | |

| Table K. Sensitivity analyses of the incident arrhythmia in patients with inflammatory bowel disease and their matched population references | | | | | |
| --- | --- | --- | --- | --- | --- |
|  | HR (95%CI) | | | | |
| Outcomes | Excluding those with COPD ^a^ | Excluding those with cardiovascular-related comorbidities ^b^ | Only individuals with available educational attainment ^a^ | Further adjusted for autoimmune thyroid disease ^c^ | Only individuals with index date later than January 2006 ^d^ |
| CD | | | | | |
| Overall arrhythmias | 1.15 (1.08, 1.21) | 1.11 (1.04, 1.19) | 1.17 (1.10, 1.24) | 1.14 (1.08, 1.21) | 1.12 (1.00, 1.26) |
| Atrial fibrillation/flutter | 1.12 (1.05, 1.19) | 1.08 (1.00, 1.17) | 1.15 (1.07, 1.23) | 1.12 (1.05, 1.19) | 1.08 (0.94, 1.23) |
| Bradyarrhythmias | 1.11 (0.95, 1.29) | 1.00 (0.83, 1.22) | 1.16 (0.99, 1.37) | 1.10 (0.94, 1.27) | 0.97 (0.69, 1.37) |
| Other supraventricular arrhythmias | 1.35 (1.14, 1.58) | 1.29 (1.08, 1.56) | 1.33 (1.11, 1.60) | 1.33 (1.13, 1.57) | 1.72 (1.23, 2.41) |
| Ventricular arrhythmias/cardiac arrest | 1.25 (1.09, 1.44) | 1.28 (1.09, 1.52) | 1.21 (1.03, 1.43) | 1.24 (1.08, 1.43) | 0.91 (0.64, 1.29) |
| UC | | | | | |
| Overall arrhythmias | 1.14 (1.10, 1.19) | 1.15 (1.09, 1.20) | 1.12 (1.08, 1.17) | 1.13 (1.09, 1.18) | 1.10 (1.01, 1.18) |
| Atrial fibrillation/flutter | 1.12 (1.07, 1.17) | 1.12 (1.07, 1.18) | 1.11 (1.06, 1.16) | 1.11 (1.07, 1.16) | 1.10 (1.01, 1.20) |
| Bradyarrhythmias | 1.03 (0.93, 1.15) | 1.05 (0.92, 1.19) | 1.03 (0.92, 1.15) | 1.03 (0.92, 1.14) | 1.02 (0.81, 1.30) |
| Other supraventricular arrhythmias | 1.32 (1.17, 1.48) | 1.27 (1.12, 1.45) | 1.31 (1.15, 1.49) | 1.31 (1.16, 1.47) | 1.28 (1.00, 1.63) |
| Ventricular arrhythmias/cardiac arrest | 1.26 (1.15, 1.39) | 1.31 (1.17, 1.47) | 1.16 (1.04, 1.30) | 1.25 (1.14, 1.37) | 1.10 (0.88, 1.37) |
| IBD-U | | | | | |
| Overall arrhythmias | 1.30 (1.19, 1.41) | 1.37 (1.22, 1.53) | 1.24 (1.14, 1.35) | 1.29 (1.19, 1.40) | 1.28 (1.13, 1.44) |
| Atrial fibrillation/flutter | 1.32 (1.20, 1.45) | 1.42 (1.25, 1.61) | 1.28 (1.16, 1.41) | 1.33 (1.21, 1.46) | 1.24 (1.08, 1.42) |
| Bradyarrhythmias | 1.23 (0.97, 1.56) | 1.45 (1.07, 1.99) | 1.20 (0.94, 1.53) | 1.21 (0.96, 1.53) | 0.99 (0.69, 1.43) |
| Other supraventricular arrhythmias | 1.32 (1.01, 1.72) | 1.14 (0.83, 1.56) | 1.28 (0.97, 1.69) | 1.36 (1.04, 1.76) | 2.21 (1.45, 3.39) |
| Ventricular arrhythmias/cardiac arrest | 1.42 (1.14, 1.77) | 1.68 (1.26, 2.23) | 1.35 (1.06, 1.70) | 1.37 (1.10, 1.70) | 1.42 (1.00, 2.04) |
| CD: Crohn's disease; CI: confidence interval; COPD: chronic obstructive pulmonary disease; HR: hazard ratio; IBD-U: inflammatory bowel disease unclassified; UC: ulcerative colitis. | | | | | |
| ^a^ Model a: Conditioned on the matching variables (birth year, sex, county of residence, and calendar year) and further adjusted for country of birth, educational attainment, number of healthcare visits, ischemic heart disease, heart failure, stroke, hypertension, diabetes, obesity, dyslipidemia, chronic kidney disease, and COPD. | | | | | |
| ^b^ Conditioned on the matching variables (birth year, sex, county of residence, and calendar year) and further adjusted for country of birth, educational attainment, and number of healthcare visits. Cardiovascular-related comorbidities include ischemic heart disease, heart failure, stroke, hypertension, diabetes, obesity dyslipidemia, chronic kidney disease, and COPD. | | | | | |
| ^c^ Model a + autoimmune thyroid disease. | | | | | |
| ^d^ Model a + further adjusted for aspirin, non-aspirin anti-platelet medications, statins, non-statin lipid lowering medications, anticoagulation medications, antidiabetic medications, and antihypertensive agents. | | | | | |

| **Table L. Incident arrhythmia in patients with inflammatory bowel disease and their matched reference individuals (1-year or 3-years lag time)** | | | | | | | | | |
| --- | --- | --- | --- | --- | --- | --- | --- | --- | --- |
|  | 1-year lag time | | | |  | 3-years lag time | | | |
| Outcomes | No. of events, n (%) | | IR (95%CI) difference, per 10,000 Pys | HR (95%CI) ^a^ |  | No. of events, n (%) | | IR (95%CI) difference, per 10,000 Pys | HR (95%CI) ^a^ |
|  | Patients | References |  |  |  | Patients | References |  |  |
| CD | | | | | | | | | |
| Overall arrhythmias | 1733 (7.2) | 7266 (6.3) | 8.0 (5.3, 10.7) | 1.11 (1.05, 1.18) |  | 1544 (6.6) | 6219 (5.7) | 9.8 (6.8, 12.8) | 1.13 (1.06, 1.20) |
| Atrial fibrillation/flutter | 1317 (5.4) | 5695 (4.9) | 4.9 (2.6, 7.3) | 1.09 (1.02, 1.16) |  | 1175 (5.1) | 4869 (4.5) | 6.3 (3.7, 8.9) | 1.10 (1.02, 1.18) |
| Bradyarrhythmias | 242 (1.0) | 1001 (0.9) | 1.2 (0.2, 2.2) | 1.08 (0.93, 1.26) |  | 219 (0.9) | 843 (0.8) | 1.6 (0.5, 2.7) | 1.11 (0.94, 1.31) |
| Other supraventricular arrhythmias | 198 (0.8) | 706 (0.6) | 1.7 (0.8, 2.5) | 1.31 (1.10, 1.55) |  | 175 (0.8) | 595 (0.5) | 1.9 (0.9, 2.9) | 1.39 (1.16, 1.67) |
| Ventricular arrhythmias/cardiac arrest | 281 (1.2) | 1055 (0.9) | 2.0 (0.9, 3.1) | 1.17 (1.01, 1.36) |  | 242 (1.0) | 879 (0.8) | 2.2 (1.0, 3.3) | 1.24 (1.05, 1.45) |
| UC | | | | | | | | | |
| Overall arrhythmias | 3823 (8.4) | 15291 (7.1) | 11.2 (9.0, 13.4) | 1.12 (1.07, 1.16) |  | 3379 (7.7) | 13028 (6.4) | 13.0 (10.6, 15.4) | 1.12 (1.07, 1.16) |
| Atrial fibrillation/flutter | 2997 (6.6) | 12070 (5.6) | 8.4 (6.5, 10.3) | 1.10 (1.05, 1.15) |  | 2658 (6.1) | 10255 (5.0) | 10.0 (7.9, 12.2) | 1.11 (1.06, 1.16) |
| Bradyarrhythmias | 493 (1.1) | 2129 (1.0) | 0.9 (0.1, 1.6) | 1.03 (0.93, 1.15) |  | 439 (1.0) | 1815 (0.9) | 1.1 (0.3, 2.0) | 1.02 (0.91, 1.15) |
| Other supraventricular arrhythmias | 360 (0.8) | 1337 (0.6) | 1.4 (0.7, 2.0) | 1.29 (1.14, 1.45) |  | 302 (0.7) | 1138 (0.6) | 1.2 (0.5, 1.9) | 1.24 (1.09, 1.42) |
| Ventricular arrhythmias/cardiac arrest | 610 (1.3) | 2151 (1.0) | 2.7 (1.8, 3.5) | 1.21 (1.09, 1.33) |  | 520 (1.2) | 1748 (0.9) | 2.9 (2.0, 3.9) | 1.23 (1.11, 1.37) |
| IBD-U | | | | | | | | | |
| Overall arrhythmias | 851 (7.4) | 2903 (5.4) | 22.4 (17.1, 27.7) | 1.24 (1.14, 1.35) |  | 669 (6.3) | 2320 (4.7) | 20.2 (14.4, 26.1) | 1.19 (1.08, 1.31) |
| Atrial fibrillation/flutter | 674 (5.9) | 2255 (4.2) | 18.3 (13.7, 23.0) | 1.27 (1.16, 1.41) |  | 520 (4.9) | 1785 (3.6) | 16.0 (10.8, 21.1) | 1.20 (1.08, 1.34) |
| Bradyarrhythmias | 107 (0.9) | 375 (0.7) | 2.5 (0.7, 4.3) | 1.18 (0.92, 1.51) |  | 83 (0.8) | 287 (0.6) | 2.4 (0.4, 4.4) | 1.22 (0.92, 1.61) |
| Other supraventricular arrhythmias | 75 (0.7) | 251 (0.5) | 2.0 (0.4, 3.5) | 1.30 (0.99, 1.72) |  | 62 (0.6) | 214 (0.4) | 1.8 (0.1, 3.5) | 1.22 (0.89, 1.66) |
| Ventricular arrhythmias/cardiac arrest | 126 (1.1) | 396 (0.7) | 3.7 (1.8, 5.7) | 1.37 (1.09, 1.72) |  | 101 (0.9) | 311 (0.6) | 3.8 (1.6, 6.0) | 1.41 (1.09, 1.82) |
| CD: Crohn's disease; CI: confidence interval; HR: hazard ratio; IBD-U: inflammatory bowel disease unclassified; IR: incidence rate; UC: ulcerative colitis; Pys: person-years. | | | | | | | | | |
| ^a^ Conditioned on the matching variables (birth year, sex, county of residence, and calendar year), and further adjusted for country of birth, educational attainment, number of healthcare visits, ischemic heart disease, heart failure, stroke, hypertension, diabetes, obesity, dyslipidemia, chronic kidney disease, and chronic obstructive pulmonary disease. | | | | | | | | | |

| **Table M. Characteristics of patients with inflammatory bowel disease and their IBD-free full siblings** | | | | | | |
| --- | --- | --- | --- | --- | --- | --- |
| Characteristics | Patients with CD, n (%) | CD siblings, n (%) | Patients with UC, n (%) | UC siblings, n (%) | Patients with IBD-U, n (%) | IBD-U siblings, n (%) |
| N | 16287 | 29631 | 30556 | 56135 | 7694 | 14018 |
| Age at index date, years ^a^ |  |  |  |  |  |  |
| Mean ± SD | 36.0 ± 16.5 | 37.3 ± 17.1 | 38.7 ± 15.7 | 40.0 ± 16.2 | 39.6 ± 18.0 | 40.8 ± 18.2 |
| Median (IQR) | 33.5 (22.2, 48.6) | 36.1 (23.3, 50.7) | 37.4 (26.2, 50.5) | 39.8 (27.3, 52.5) | 38.1 (24.7, 54.3) | 40.7 (25.8, 55.3) |
| <18 | 2317 (14.2) | 3674 (12.4) | 2551 (8.4) | 4126 (7.4) | 940 (12.2) | 1519 (10.8) |
| 18-39 | 7668 (47.1) | 13397 (45.2) | 14356 (47.0) | 24641 (43.9) | 3133 (40.7) | 5423 (38.7) |
| 40-59 | 4717 (29.0) | 9450 (31.9) | 10290 (33.7) | 20582 (36.7) | 2354 (30.6) | 4636 (33.1) |
| ≥60 | 1585 (9.7) | 3110 (10.5) | 3359 (11.0) | 6786 (12.1) | 1267 (16.5) | 2440 (17.4) |
| Female | 8367 (51.4) | 14551 (49.1) | 14067 (46.0) | 27722 (49.4) | 3721 (48.4) | 7015 (50.0) |
| Born in Nordic country ^b^ | 15746 (96.7) | 28326 (95.6) | 29900 (97.9) | 54670 (97.4) | 7504 (97.5) | 13511 (96.4) |
| Calendar period at index date ^a^ |  |  |  |  |  |  |
| 1969-1989 | 1243 (7.6) | 2457 (8.3) | 1696 (5.6) | 3448 (6.1) | 171 (2.2) | 335 (2.4) |
| 1990-1999 | 3569 (21.9) | 6603 (22.3) | 6544 (21.4) | 12692 (22.6) | 1014 (13.2) | 1975 (14.1) |
| 2000-2009 | 6528 (40.1) | 11872 (40.1) | 13439 (44.0) | 24535 (43.7) | 2909 (37.8) | 5329 (38.0) |
| 2010-2019 | 4947 (30.4) | 8699 (29.4) | 8877 (29.1) | 15460 (27.5) | 3600 (46.8) | 6379 (45.5) |
| Educational attainment, years |  |  |  |  |  |  |
| 0-9 | 3336 (20.5) | 6378 (21.5) | 5545 (18.2) | 11567 (20.6) | 1512 (19.7) | 2816 (20.1) |
| 10-12 | 6575 (40.4) | 11374 (38.4) | 13085 (42.8) | 23267 (41.5) | 3218 (41.8) | 5897 (42.1) |
| ≥13 | 3404 (20.9) | 5992 (20.2) | 8290 (27.1) | 13518 (24.1) | 1924 (25.0) | 3336 (23.8) |
| Missing | 2972 (18.3) | 5887 (19.9) | 3636 (11.9) | 7783 (13.9) | 1040 (13.5) | 1969 (14.1) |
| Number of healthcare visits ^c^ |  |  |  |  |  |  |
| 0 | 9396 (57.7) | 21948 (74.1) | 19332 (63.3) | 42312 (75.4) | 3967 (51.6) | 9625 (68.7) |
| 1 | 2675 (16.4) | 3692 (12.5) | 4955 (16.2) | 6695 (11.9) | 1327 (17.3) | 1966 (14.0) |
| 2-3 | 2128 (13.1) | 2394 (8.1) | 3555 (11.6) | 4377 (7.8) | 1188 (15.4) | 1431 (10.2) |
| ≥4 | 2088 (12.8) | 1597 (5.4) | 2714 (8.9) | 2751 (4.9) | 1212 (15.8) | 996 (7.1) |
| Disease history before index date ^a^ |  |  |  |  |  |  |
| Ischemic heart disease | 727 (4.5) | 1105 (3.7) | 1282 (4.2) | 1974 (3.5) | 524 (6.8) | 677 (4.8) |
| Heart failure | 102 (0.6) | 126 (0.4) | 155 (0.5) | 206 (0.4) | 94 (1.2) | 77 (0.6) |
| Stroke | 429 (2.6) | 587 (2.0) | 707 (2.3) | 1136 (2.0) | 345 (4.5) | 404 (2.9) |
| Hypertension | 1234 (7.6) | 1627 (5.5) | 1998 (6.5) | 2987 (5.3) | 976 (12.7) | 1168 (8.3) |
| Diabetes | 704 (4.3) | 1172 (4.0) | 1348 (4.4) | 2089 (3.7) | 545 (7.1) | 713 (5.1) |
| Obesity | 259 (1.6) | 357 (1.2) | 276 (0.9) | 543 (1.0) | 167 (2.2) | 209 (1.5) |
| Dyslipidemia | 391 (2.4) | 583 (2.0) | 751 (2.5) | 1144 (2.0) | 304 (4.0) | 428 (3.1) |
| Chronic kidney disease | 71 (0.4) | 52 (0.2) | 103 (0.3) | 91 (0.2) | 82 (1.1) | 42 (0.3) |
| COPD | 221 (1.4) | 247 (0.8) | 332 (1.1) | 452 (0.8) | 166 (2.2) | 169 (1.2) |
| Montreal Classification CD at index date ^d^ |  |  |  |  |  |  |
| L1, L3/LX (Ileal, ileocolonic or location not defined) | 10010 (61.5) | - | - | - | - | - |
| L2 (Colonic) | 2424 (14.9) | - | - | - | - | - |
| Perianal | 917 (5.6) | - | - | - | - | - |
| Montreal Classification UC at index date ^e^ |  |  |  |  |  |  |
| E1/E2 (Proctitis, left-sided colitis) | - | - | 8902 (29.1) | - | - | - |
| E3 (Extensive colitis) | - | - | 5057 (16.6) | - | - | - |
| EX (Extent not defined) | - | - | 10121 (33.1) | - | - | - |
| Extraintestinal manifestations |  |  |  |  |  |  |
| Primary sclerosing cholangitis | 99 (0.6) | - | 534 (1.8) | - | 131 (1.7) | - |
| Other extraintestinal manifestations | 985 (6.1) | - | 1129 (3.7) | - | 524 (6.8) | - |
| Follow-up time, years |  |  |  |  |  |  |
| Median (IQR) | 13.7 (7.7, 20.2) | 13.9 (7.8, 20.5) | 13.9 (8.0, 19.4) | 14.1 (8.1, 19.8) | 9.7 (5.6, 15.6) | 9.9 (6.0, 16.1) |
| 0-0.9 | 256 (1.6) | 405 (1.4) | 448 (1.5) | 704 (1.3) | 241 (3.1) | 329 (2.4) |
| 1-4.9 | 1671 (10.3) | 3012 (10.2) | 2847 (9.3) | 5197 (9.3) | 1371 (17.8) | 2305 (16.4) |
| 5-9.9 | 3781 (23.2) | 6817 (23.0) | 6972 (22.8) | 12544 (22.4) | 2366 (30.8) | 4405 (31.4) |
| 10-19.9 | 6423 (39.4) | 11625 (39.2) | 13200 (43.2) | 23909 (42.6) | 2750 (35.7) | 5059 (36.1) |
| ≥20 | 4156 (25.5) | 7772 (26.2) | 7089 (23.2) | 13781 (24.6) | 966 (12.6) | 1920 (13.7) |
| CD, Crohn’s disease; COPD, chronic obstructive pulmonary disease; E, Extent; IBD-U: inflammatory bowel disease unclassified; IQR, interquartile range; L, location; SD, standard deviation; UC, ulcerative colitis. | | | | | | |
| ^a^ Index date: date of IBD diagnosis for patients, and date of selection for their IBD-free full siblings. | | | | | | |
| ^b^ Nordic country includes Sweden, Denmark, Finland, Norway, and Iceland. | | | | | | |
| ^c^ Defined as the number of healthcare visits between 2 years and 6 months before the index date. | | | | | | |
| ^d^ Not every patient with CD has the location information. | | | | | | |
| ^e^ Not every patient with UC has extent information. | | | | | | |

| **Table N. Incident arrhythmia in patients with inflammatory bowel disease and their IBD-free full siblings** | | | | | | | |
| --- | --- | --- | --- | --- | --- | --- | --- |
| Outcomes | No. of events, n (%) | | IR (95%CI), per 10,000 Pys | | IR (95%CI) difference, per 10,000 Pys | HR (95%CI) | |
|  | Patients | References | Patients | References |  | Model 1 ^a^ | Model 2 ^b^ |
| CD | | | | | | | |
| Overall arrhythmias | 862 (5.3) | 1681 (5.7) | 35.8 (33.5, 38.3) | 37.9 (36.2, 39.8) | -2.1 (-5.1, 0.9) | 1.15 (1.03, 1.28) | 1.09 (0.97, 1.22) |
| Atrial fibrillation/flutter | 616 (3.8) | 1251 (4.2) | 25.5 (23.5, 27.5) | 28.1 (26.5, 29.7) | -2.6 (-5.2, -0.1) | 1.12 (0.99, 1.28) | 1.07 (0.93, 1.22) |
| Bradyarrhythmias | 120 (0.7) | 228 (0.8) | 4.9 (4.1, 5.9) | 5.1 (4.4, 5.8) | -0.1 (-1.2, 0.9) | 1.10 (0.84, 1.45) | 0.94 (0.69, 1.28) |
| Other supraventricular arrhythmias | 137 (0.8) | 197 (0.7) | 5.6 (4.7, 6.6) | 4.4 (3.8, 5.0) | 1.2 (0.1, 2.4) | 1.35 (1.04, 1.77) | 1.39 (1.04, 1.86) |
| Ventricular arrhythmias/cardiac arrest | 130 (0.8) | 256 (0.9) | 5.3 (4.4, 6.3) | 5.7 (5.0, 6.4) | -0.4 (-1.5, 0.8) | 1.12 (0.86, 1.46) | 0.95 (0.71, 1.29) |
| UC | | | | | | | |
| Overall arrhythmias | 1841 (6.0) | 3218 (5.7) | 41.5 (39.6, 43.4) | 38.8 (37.5, 40.2) | 2.7 (0.3, 5.0) | 1.22 (1.13, 1.31) | 1.18 (1.09, 1.28) |
| Atrial fibrillation/flutter | 1378 (4.5) | 2436 (4.3) | 30.9 (29.2, 32.5) | 29.2 (28.1, 30.4) | 1.6 (-0.4, 3.6) | 1.22 (1.11, 1.33) | 1.19 (1.09, 1.30) |
| Bradyarrhythmias | 224 (0.7) | 464 (0.8) | 4.9 (4.3, 5.6) | 5.5 (5.0, 6.0) | -0.5 (-1.4, 0.3) | 0.99 (0.80, 1.21) | 0.92 (0.74, 1.14) |
| Other supraventricular arrhythmias | 240 (0.8) | 395 (0.7) | 5.3 (4.7, 6.0) | 4.7 (4.2, 5.2) | 0.6 (-0.2, 1.4) | 1.24 (1.01, 1.51) | 1.20 (0.97, 1.47) |
| Ventricular arrhythmias/cardiac arrest | 282 (0.9) | 430 (0.8) | 6.2 (5.5, 7.0) | 5.1 (4.6, 5.6) | 1.1 (0.3, 2.0) | 1.33 (1.10, 1.61) | 1.27 (1.04, 1.56) |
| IBD-U | | | | | | | |
| Overall arrhythmias | 445 (5.8) | 679 (4.8) | 51.7 (47.0, 56.7) | 41.9 (38.8, 45.2) | 9.8 (4.1, 15.5) | 1.25 (1.05, 1.48) | 1.19 (0.99, 1.42) |
| Atrial fibrillation/flutter | 333 (4.3) | 489 (3.5) | 38.5 (34.5, 42.8) | 30.0 (27.4, 32.8) | 8.5 (3.6, 13.4) | 1.32 (1.08, 1.60) | 1.24 (1.01, 1.53) |
| Bradyarrhythmias | 56 (0.7) | 94 (0.7) | 6.4 (4.8, 8.3) | 5.7 (4.6, 7.0) | 0.7 (-1.4, 2.7) | 1.31 (0.82, 2.10) | 1.37 (0.82, 2.30) |
| Other supraventricular arrhythmias | 54 (0.7) | 88 (0.6) | 6.1 (4.6, 8.0) | 5.3 (4.3, 6.6) | 0.8 (-1.2, 2.8) | 0.95 (0.60, 1.49) | 0.90 (0.52, 1.55) |
| Ventricular arrhythmias/cardiac arrest | 69 (0.9) | 99 (0.7) | 7.8 (6.1, 9.9) | 6.0 (4.9, 7.3) | 1.8 (-0.4, 4.0) | 1.65 (1.06, 2.55) | 1.56 (0.96, 2.54) |
| CD: Crohn's disease; CI: confidence interval; HR: hazard ratio; IBD-U: inflammatory bowel disease unclassified; IR: incidence rate; UC: ulcerative colitis; Pys: person-years. | | | | | | | |
| ^a^ Conditioned on family identifier and adjusted for birth year, sex, county of residence, and calendar year. | | | | | | | |
| ^b^ Further adjusted for country of birth, educational attainment, number of healthcare visits, ischemic heart disease, heart failure, stroke, hypertension, diabetes, obesity, dyslipidemia, chronic kidney disease, and chronic obstructive pulmonary disease. | | | | | | | |

**Reference**

1. Mubasher M, Syed T, Hanafi A, Yu Z, Yusuf I, Abdullah AS, et al. An Investigation into the Association Between Inflammatory Bowel Disease and Cardiac Arrhythmias: An Examination of the United States National Inpatient Sample Database. Clin Med Insights Cardiol. 2020;14:1179546820955179. doi: 10.1177/1179546820955179. PMID: 33192109.

2. Choi YJ, Choi EK, Han KD, Park J, Moon I, Lee E, et al. Increased risk of atrial fibrillation in patients with inflammatory bowel disease: A nationwide population-based study. World J Gastroenterol. 2019;25(22):2788-98. doi: 10.3748/wjg.v25.i22.2788. PMID: 31236001.

3. Pattanshetty DJ, Anna K, Gajulapalli RD, Sappati-Biyyani RR. Inflammatory bowel "Cardiac" disease: Point prevalence of atrial fibrillation in inflammatory bowel disease population. Saudi J Gastroenterol. 2015;21(5):325-9. doi: 10.4103/1319-3767.166208. PMID: 26458861.

4. Kristensen SL, Lindhardsen J, Ahlehoff O, Erichsen R, Lamberts M, Khalid U, et al. Increased risk of atrial fibrillation and stroke during active stages of inflammatory bowel disease: a nationwide study. Europace. 2014;16(4):477-84. doi: 10.1093/europace/eut312. PMID: 24108228.

5. Nguyen LH, Örtqvist AK, Cao Y, Simon TG, Roelstraete B, Song M, et al. Antibiotic use and the development of inflammatory bowel disease: a national case-control study in Sweden. The Lancet Gastroenterology & Hepatology. 2020;5(11):986-95. doi: 10.1016/s2468-1253(20)30267-3.

6. Mouratidou N, Malmborg P, Jaras J, Sigurdsson V, Sandstrom O, Fagerberg UL, et al. Identification of Childhood-Onset Inflammatory Bowel Disease in Swedish Healthcare Registers: A Validation Study. Clin Epidemiol. 2022;14:591-600. doi: 10.2147/CLEP.S358031. PMID: 35520278.

7. Everhov AH, Sachs MC, Malmborg P, Nordenvall C, Myrelid P, Khalili H, et al. Changes in inflammatory bowel disease subtype during follow-up and over time in 44,302 patients. Scand J Gastroenterol. 2019;54(1):55-63. doi: 10.1080/00365521.2018.1564361. PMID: 30700170.

8. Shrestha S, Olen O, Eriksson C, Everhov AH, Myrelid P, Visuri I, et al. The use of ICD codes to identify IBD subtypes and phenotypes of the Montreal classification in the Swedish National Patient Register. Scand J Gastroenterol. 2020;55(4):430-5. doi: 10.1080/00365521.2020.1740778. PMID: 32370571.

9. Olén O, Erichsen R, Sachs MC, Pedersen L, Halfvarson J, Askling J, et al. Colorectal cancer in ulcerative colitis: a Scandinavian population-based cohort study. The Lancet. 2020;395(10218):123-31. doi: 10.1016/s0140-6736(19)32545-0.
