# Supplementary material for: Long-term risk of arrhythmias in patients with inflammatory bowel disease: A population-based, sibling-controlled cohort study
Source: PLoS Med. 2023 Oct 19;20(10):e1004305. doi: 10.1371/journal.pmed.1004305 (PMC10621936; doi:10.1371/journal.pmed.1004305)
Supplement: S2 Appendix — (DOCX) [file pmed.1004305.s003.docx]

**Long-term risk of arrhythmias in patients with inflammatory bowel disease: A population-based, sibling-controlled cohort study**

J Sun et al.

**Flexible parametric survival model vs. Cox regression model**

Through using restricted cubic spline functions to model the baseline cumulative hazard or some other general baseline distribution in the survival analysis model, flexible parametric survival models enable proportional hazards, proportional odds and probit models to be fitted [1]. Compared with the Cox regression model, flexible parametric survival models are easy to make smooth predictions (e.g., providing smooth estimates of the hazard and survival functions for any combination of covariate values), to model complicated time-dependent effects (e.g., getting any type of time-dependent hazard ratios), to model multiple time scales (i.e., in a Cox model, we usually consider only one time scale), to investigate absolute and relative effects, and to incorporate expected mortality for the relative survival analysis [2].

Reference

1. Lambert PC, Royston P. Further development of flexible parametric models for survival analysis. Stata J. 2009;9(2):265-90. doi: Doi 10.1177/1536867x0900900206. PMID: WOS:000268973400006.

2. Royston P, Lambert PC. Flexible parametric survival analysis using Stata: beyond the Cox model: Stata press College Station, TX; 2011.
