# Supplementary material for: Long-term risk of arrhythmias in patients with inflammatory bowel disease: A population-based, sibling-controlled cohort study
Source: PLoS Med. 2023 Oct 19;20(10):e1004305. doi: 10.1371/journal.pmed.1004305 (PMC10621936; doi:10.1371/journal.pmed.1004305)
Supplement: S1 Text — (DOCX) [file pmed.1004305.s004.docx]

**Statistical Analysis Plan**

**Long-term risk of arrhythmias in patients with inflammatory bowel disease: A population-based, sibling-controlled cohort study**

Jiangwei Sun, PhD

[Jiangwei.sun@ki.se](mailto:Jiangwei.sun@ki.se)

Jonas F. Ludvigsson, MD, PhD

[jonasludvigsson@yahoo.com](mailto:jonasludvigsson@yahoo.com)

Department of Medical Epidemiology and Biostatistics

Karolinska Institutet

Stockholm

Sweden

**MODIFICATION HISTOY:**

| Version(Date) | Authors | Action/modification |
| --- | --- | --- |
| V1 (2022-02-21) | Jiangwei Sun, Jonas F. Ludvigsson | Created this statistical analysis plan (SAP) |
| V2 (2022-10-12) | Jiangwei Sun, Jonas F. Ludvigsson | Revised SAP based on all coauthors' comments and the analyses will be conducted after this version until January 2023. |
| V3 (2023-07-07) | Jiangwei Sun, Jonas F. Ludvigsson | Revised SAP based on comments from external reviewers |

**COAUTHORS:**

IBD experts: Jonas Halfvarson, Anders Forss, Ola Olén

Cardiology or arrhythmia expert: Emma Svennberg, Johan Sundström

Biostatistician: Bjorn Roelstraete

**STUDY OBJECTIVES**

To investigate the long-term risk of arrhythmias in patients with inflammatory bowel disease (IBD).

**HYPOTHESIS**

Because of the present knowledge about the role of systemic inflammation in the development of cardiovascular diseases, we hypothesize that patients with IBD are at an increased risk of overall arrhythmias and specific types of arrhythmias, including atrial fibrillation/flutter, bradyarrhythmias, other supraventricular arrhythmias, and ventricular arrhythmias/cardiac arrest.

**METHODS**

**Data source, study design and participants**

This cohort will be based on the nationwide histopathology cohort, Epidemiology Strengthened by histoPathology Reports in Sweden (ESPRESSO) [1], and several healthcare registers in Sweden. ESPRESSO includes histopathology data from all 28 pathology departments in Sweden recorded between 1969 to 2019 (changed into 2017 in the revision), including date on biopsy, topography, and morphology (recorded by the Swedish version of the Systematized Nomenclature of Medicine (SNOMED) system) [1].

Patients with IBD will be identified as those with at least one International Classification of Disease (ICD) code for IBD in the Swedish National Patient Register (NPR) and one biopsy record indicating IBD in the ESPRESSO (see below **Table 1** for ICD codes, SNOMED codes, and definitions of IBD subtypes)[2]. Date of IBD diagnosis (i.e., index date) will be defined as the second of receiving first ICD or first biopsy codes.

The Montreal classification will be used to define Crohn's disease (CD) location, ulcerative colitis (UC) extent, and perianal disease modifier [3]. CD location includes ileal (L1)/ileocolonic (L3)/unknown (LX) or colonic (L2). UC extent includes proctitis (E1)/left-sided colitis (E2), extensive colitis (E3) or extent not defined (EX) (see below **Table 2** for ICD codes).

**General population reference individuals and sibling comparators**

For each patient with IBD, we will randomly select up to five reference individuals from the Total Population Register (matched by birth year, sex, county of residence, and calendar year)[4]. Full siblings to patients with IBD will be identified through the Multi-Generation Register [5]. The reference individuals and full siblings have to be alive, living in Sweden, and free of IBD and arrhythmia when being selected (i.e., index date).

**Follow-up and ascertainment of outcome**

Individuals with previous arrhythmia (see below **Table 3** for ICD codes) before index date will be excluded. Follow-up will start at index date until incident diagnosis of arrhythmias, emigration, death, or December 31, 2019, whichever occurs first. Reference individuals or IBD-free full siblings will also be censored when receiving a diagnosis of IBD during follow-up. Individuals with an incident arrhythmia will be identified from the NPR, considering both primary and secondary diagnosis. The primary outcome is the incidence of overall arrhythmias. The secondary outcomes are the incidences of atrial fibrillation/flutter, bradyarrhythmias, other supraventricular arrhythmias, and ventricular arrhythmias/cardiac arrest. If one individual is diagnosed with more than one of the secondary outcomes, this individual will contribute to each outcome with the respective diagnosis date.

**Covariates**

We will retrieve information on country of birth (available from 1947 onward, Nordic or others) from the Total Population Register [4] and educational attainment (available from 1990 onward; 4 groups: 0-9 y, 10-12 y, ≥13 y, and “missing”, a proxy for socioeconomic status) from the Swedish Longitudinal Integrated Database for Health Insurance and Labour Market Studies [6]. As a proxy for regular healthcare seeking behavior, number of healthcare visits will be retrieved from the NPR (4 groups: 0, 1, 2-3, and ≥4,) which is defined as the number of specialized (non-primary care) outpatient visits or hospitalizations between 2 years and 6 months before index date. Data on cardiovascular related comorbidities preceding index date will be collected from the NPR, including ischemic heart disease, heart failure, stroke, hypertension, diabetes, obesity dyslipidemia, chronic kidney disease, and chronic obstructive pulmonary disease (COPD, only if patient diagnosed ≥40 year) (see below **Table 4** for ICD codes). Information regarding use of the cardiovascular related medications preceding index date will be collected from the Prescribed Drug Register [7] (data available from July 2005 onward): aspirin, non-aspirin anti-platelet medications, statins, non-statin lipid lowering medications, anticoagulation medications, antidiabetic medications, and antihypertensive agents (see below **Table 5** for the Anatomical Therapeutic Chemical codes).

**STATISTICAL ANALYSES**

We will report incidence rate (IR) and IR difference, together with 95% confidence intervals (CIs) for all outcomes. To estimate the average and temporal patterns of hazard ratios (HRs) and 95% CIs for incident arrhythmias in relation to IBD, flexible parametric survival models will be applied to allow the effect of IBD to vary over time (time-varying effect)[8]. Time since index date will be used as the underlying time scale. Standardized cumulative incidence and its differences of arrhythmia will be estimated. We will report the cumulative incidence difference at 1 year, 5 years, 10 years, and 25 years after index date. In the population matched cohort, we will condition the analyses on the matching variables (birth year, sex, county of residence, and calendar year) in model 1, and additionally adjust for country of birth, educational attainment, number of healthcare visits, ischemic heart disease, heart failure, stroke, hypertension, diabetes, obesity, dyslipidemia, chronic kidney disease, and COPD in model 2.

**Subgroup and sensitivity analyses**

We will calculate the risk of overall arrhythmia by sex, age at index date (<18, 18-<40, 40-<60, and ≥60), calendar period at index date (1969-1989, 1990-1999, 2000-2009, and 2010-2019), educational attainment (0-9 y, 10-12 y, ≥13 y, and “missing”), and number of healthcare visits (0, 1, 2-3, and ≥4). We will also explore the associations by location for CD, by extent for UC according to the Montreal classification, and by occurrence of primary sclerosing cholangitis or other extraintestinal manifestations (see below **Table 2** for ICD codes) [3].

Several sensitivity analyses will be conducted. **First,** due to lack of detailed data on smoking, we will restrict the analysis to those without COPD (a proxy for heavy smoking) before index date. **Second,** we will restrict the analysis to individuals with data available on educational attainment. **Third,** given that the Prescribed Drug Register is available since July 2005, we will restrict the analysis to individuals with index date later than 1 January 2006 and apply the statistical models further adjusting for the above mentioned cardiovascular related medications. **Fourth,** we will repeat the main analysis after excluding individuals with incident arrhythmias recorded within 1 year or within 3 years of follow-up after index date. **Fifth,** we will compare patients with IBD with their IBD-free full siblings after conditioning on family identifier and adjusting for birth year, sex, county of residence, calendar year, and all above mentioned covariates.

Data analyses will be performed using SAS version 9.4 (SAS Institute Inc, Cary, NC), Stata (version 16.1; Stata Corp LP, College Station, TX), and R version 3.6.0. A two-sided *P* ≤ 0.05 will be considered statistically significant.

**Ethics consideration**

This study is approved by the Stockholm Ethics Review Board (2014/1287-31/4 and 2018/972-32).

*Non-prespecified Analyses (Analyses according to comments from external reviewers):*

- To assess the influence of cardiovascular-related comorbidities on the associations, we excluded those with cardiovascular-related comorbidities before the index date from the analysis.
- In a sensitivity analysis, we further adjusted for autoimmune thyroid disease in the model to assess its influence on the associations.

| **Table 1. International Classification of Disease (ICD) codes and SNOMED codes defining inflammatory bowel disease (IBD) ^a^**. | | | | | |
| --- | --- | --- | --- | --- | --- |
|  | ICD-7 (1964-1968) | ICD-8 (1969-1986) | ICD-9 (1987-1996) | ICD-10 (1997-) | SNOMED codes ^b^ |
| Ulcerative colitis (UC) | 572,20; 572,21; 578,03 | 563,1; 563,10; 569,02; 569,04 | 556 | K51 | D6255 or M41, M42, M43, M44, M463, or M47 |
| Crohn’s disease (CD) | 572,00; 572,09 | 563,00 | 555 | K50 | D6216 or M41, M42, M43, M44, M463, or M47 |
| IBD unclassified (IBD-U) | UC + CD | UC + CD or 563; 563,0; 563,9; 563,98; 563,99 | UC + CD | K52.3 | D6214 or M41, M42, M43, M44, M463, or M47 |
| ^a^ ≥1 ICD code for IBD plus a relevant biopsy code has a positive predictive value of 95% [9,10]. | | | | | |
| ^b^ D codes are diagnostic codes but listed under morphology in pathology registers; D6255 for example is the diagnostic code for UC. SNOMED codes starting with "M"; M41 for example refers to all codes starting with "M41". | | | | | |
| Because definitions of exposure should not “look into the future”, IBD subtypes were defined in accordance with the first two diagnostic codes only (i.e., no information after start of follow-up contributed to the subtype definition). For individuals with one ICD code for IBD and one unspecific SNOMED code (i.g., “M…”), the IBD subtype was determined by the ICD code in the patient register only. In a recent paper [11], we report that 18% of incident patients with IBD in the Swedish patient register during 2002-2014 were classified as another IBD subtype at some point during follow-up. | | | | | |

| **Table 2. ICD codes assigned for phenotypes of inflammatory bowel disease** | |
| --- | --- |
| Montreal classification | Diagnostic codes |
| Crohn’s disease location ^a^ |  |
| Ileal (L1) | K50.0 |
| Colonic (L2) | K50.1 |
| Ileocolonic or location not defined (L3/LX) | K50.8, K50.9 |
| Ulcerative colitis extent ^a^ |  |
| Proctitis (E1) | K51.2 |
| Left-sided colitis (E2) | K51.3; K51.5 |
| Extensive colitis (E3) | K51.0 |
| Extent not defined (EX) | K51.4; K51.8; K51.9 |
| Primary sclerosing cholangitis ^b^ | ICD-9 (1987-1996): 576B |
|  | ICD-10 (1997-): K830 |
| Other extraintestinal manifestations | ICD-9: 695C, 364, 713B, 720A, 720C, 720W, 720X |
|  | ICD-10: L52, L88, L98.2, H20, M07.4, M07.5, M07.6, M09.1, M09.2, M45, M460, M461,, M468, M469, M139, M255 |
| ^a^ Validated definitions and diagnostic codes [3] used to define Crohn’s disease and ulcerative colitis according to the Montreal classification since the start of use of the ICD-10 in Sweden (1997-). All codes are captured in the Swedish National Patient Register (prospectively recorded in routine clinical practice). | |
| ^b^ We restricted our use to ICD-9 and ICD-10 codes since we believe that earlier ICD codes for extraintestinal inflammation are less reliable, particularly for primary sclerosing cholangitis (PSC). The validity of the PSC codes has not formally been tested in Sweden. But according to Professor Annika Bergquist (annika.bergquist@ki.se, a renowned researcher in PSC epidemiology at Karolinska Institutet and also the PI for a large Swedish PSC-cohort and biobank), the impression is that the sensitivity and positive predictive value of K830A for identifying PSC in IBD in the National Patient Register is not perfect, but the nationwide prevalence of PSC in IBD in the National Patient Register is roughly as would be expected, based on the prevalence in detailed patient cohorts at the university hospitals. | |

| **Table 3. Definitions of primary and secondary outcomes** | |
| --- | --- |
| Outcome | Definition |
| Primary outcome |  |
| Overall arrhythmia | Composite outcome including any individual outcomes from 1a-1d. |
| Secondary outcomes |  |
| 1a. Atrial fibrillation/flutter | ICD-8: 427,92 |
|  | ICD-9: 427D |
|  | ICD-10: I48 |
| 1b. Bradyarrhythmias | ICD-8: 427,20; 427,27; 427,28; 427,29 |
|  | ICD-9: 426A; 426B; 426G; 426X |
|  | ICD-10: I441; I442; I452; I453; I459; I495 |
| 1c. Other supraventricular arrhythmias | ICD-8: 427,90 |
|  | ICD-9: 426H; 427A |
|  | ICD-10: I456; I471 |
| 1d. Ventricular arrhythmias/cardiac arrest | ICD-8: 427,91; 795,99 |
|  | ICD-9: 427B; 427E; 427F; 798B; 798C |
|  | ICD-10: I460; I461; I469; I470; I472; I490; R960 |

| **Table 4. Definitions of comorbidities** | |
| --- | --- |
| **Comorbidity** | **Definition** |
| Ischemic heart disease | ICD-8: 410-414 |
|  | ICD-9: 410-414 |
|  | ICD-10: I20-25 |
| Heart failure | ICD-8: 428 |
|  | ICD-9: 428 |
|  | ICD-10: I42; I50 |
| Stroke | ICD-8: 431-434 |
|  | ICD-9: 431-434 |
|  | ICD-10: I61-64 |
| Hypertension | ICD-8: 400-404 |
|  | ICD-9: 401-405 |
|  | ICD-10: I10-I15 |
|  | Medication ATC codes: see antihypertensive medications in Table 5 |
| Obesity | ICD-8: 277 |
|  | ICD-9: 278A, 278B |
|  | ICD-10: E65-66 |
| Diabetes | ICD-8: 250 |
|  | ICD-9: 250 |
|  | ICD-10: E10-E14, O24 |
|  | Medication ATC codes: see antidiabetic medications in Table 5 |
| Dyslipidemia | ICD-8: 279 |
|  | ICD-9: 272 |
|  | ICD-10: E78 |
|  | Medication ATC codes: C10 |
| Chronic kidney disease | ICD-8: 585, 586, Y29,01 |
|  | ICD-9: 585, 586, 753B, V42A, V45B, V56 |
|  | ICD-10: N18, N19; N26; T824; Y841; Q61; Z49; Z992; Z940 |
| Chronic obstructive pulmonary disease (COPD)  (only if patient diagnosed ≥40 year) | ICD-8: 491, 492 |
|  | ICD-9: 491, 492, 496 |
|  | ICD-10: J41-J44 |

| **Table 5. Definitions of prescription medications** | |
| --- | --- |
| **Covariates** | **Definition** |
| Aspirin | B01AC06 |
| Non-aspirin anti-platelet medications | B01AC excluding aspirin (B01AC06) |
| Statins | C10AA |
| Non-statin lipid lowering medications | C10AB, C10AC, C10AD, C10AX01-14 |
| Anticoagulation medications | B01AA, B01AE, B01AF, B01AX |
| Antidiabetic medications | A10 |
| Antihypertensive medications | C02, C03AA-AB, C03BA, C03CA, C03DA, C03EA, C08CA, C08DA, C08DB, C09A, C09BA, C09BB, C09CA, C09DA, C09DB01 |

**Reference:**

1. Ludvigsson JF, Lashkariani M. Cohort profile: ESPRESSO (Epidemiology Strengthened by histoPathology Reports in Sweden). Clin Epidemiol. 2019;11:101-14. doi: 10.2147/CLEP.S191914. PMID: 30679926.

2. Forss A, Clements M, Bergman D, Roelstraete B, Kaplan GG, Myrelid P, et al. A nationwide cohort study of the incidence of inflammatory bowel disease in Sweden from 1990 to 2014. Aliment Pharmacol Ther. 2022;55(6):691-9. doi: 10.1111/apt.16735. PMID: 34907544.

3. Shrestha S, Olen O, Eriksson C, Everhov AH, Myrelid P, Visuri I, et al. The use of ICD codes to identify IBD subtypes and phenotypes of the Montreal classification in the Swedish National Patient Register. Scand J Gastroenterol. 2020;55(4):430-5. doi: 10.1080/00365521.2020.1740778. PMID: 32370571.

4. Ludvigsson JF, Almqvist C, Bonamy AK, Ljung R, Michaelsson K, Neovius M, et al. Registers of the Swedish total population and their use in medical research. Eur J Epidemiol. 2016;31(2):125-36. doi: 10.1007/s10654-016-0117-y. PMID: 26769609.

5. Ekbom A. The Swedish multi-generation register. Methods in biobanking: Springer; 2011. p. 215-20.

6. Ludvigsson JF, Svedberg P, Olen O, Bruze G, Neovius M. The longitudinal integrated database for health insurance and labour market studies (LISA) and its use in medical research. Eur J Epidemiol. 2019;34(4):423-37. doi: 10.1007/s10654-019-00511-8. PMID: 30929112.

7. Wettermark B, Hammar N, Fored CM, Leimanis A, Otterblad Olausson P, Bergman U, et al. The new Swedish Prescribed Drug Register--opportunities for pharmacoepidemiological research and experience from the first six months. Pharmacoepidemiol Drug Saf. 2007;16(7):726-35. doi: 10.1002/pds.1294. PMID: 16897791.

8. Lambert PC, Royston P. Further development of flexible parametric models for survival analysis. Stata J. 2009;9(2):265-90. doi: Doi 10.1177/1536867x0900900206. PMID: WOS:000268973400006.

9. Nguyen LH, Örtqvist AK, Cao Y, Simon TG, Roelstraete B, Song M, et al. Antibiotic use and the development of inflammatory bowel disease: a national case-control study in Sweden. The Lancet Gastroenterology & Hepatology. 2020;5(11):986-95. doi: 10.1016/s2468-1253(20)30267-3.

10. Mouratidou N, Malmborg P, Jaras J, Sigurdsson V, Sandstrom O, Fagerberg UL, et al. Identification of Childhood-Onset Inflammatory Bowel Disease in Swedish Healthcare Registers: A Validation Study. Clin Epidemiol. 2022;14:591-600. doi: 10.2147/CLEP.S358031. PMID: 35520278.

11. Everhov AH, Sachs MC, Malmborg P, Nordenvall C, Myrelid P, Khalili H, et al. Changes in inflammatory bowel disease subtype during follow-up and over time in 44,302 patients. Scand J Gastroenterol. 2019;54(1):55-63. doi: 10.1080/00365521.2018.1564361. PMID: 30700170.
