# Supplementary material for: Long-term risk of arrhythmias in patients with inflammatory bowel disease: A population-based, sibling-controlled cohort study
Source: PLoS Med. 2023 Oct 19;20(10):e1004305. doi: 10.1371/journal.pmed.1004305 (PMC10621936; doi:10.1371/journal.pmed.1004305)
Supplement: Attachment — Submitted filename: R1_letter to editor and reviewers_arrhythmias_final.docx [file pmed.1004305.s005.docx]

**Requests from the editors:**

**GENERAL COMMENTS**

Please respond to all editor and reviewer comments.

**Reply:** Done.

Please cite your Supporting Information as outlined here (<https://journals.plos.org/plosmedicine/s/supporting-information> ).

**Reply:** Done.

Please ensure that the study is reported according to the STROBE guideline, and include the completed STROBE checklist as Supporting Information. Please add the following statement, or similar, to the Methods: "This study is reported as per the Strengthening the Reporting of Observational Studies in Epidemiology (STROBE) guideline (S1 Checklist)."When completing the checklist, please use section and paragraph numbers, rather than page numbers.

**Reply:** We appreciate your suggestion and have now added the STROBE checklist accordingly, referred to it in the Methods, and attached it as a supplementary file (S1 Checklist).

On page 12 (page number in the version with track changes):

*“This study is reported as per the Strengthening the Reporting of Observational Studies in Epidemiology (STROBE) guideline (S1 Checklist)”*

Did your study have a prospective protocol or analysis plan? Please state this (either way) early in the Methods section.

**Reply:** Thank you for the suggestion. We have mentioned our pre-specified analysis plan in Method, added it as a supplementary file (S1 Text), and highlighted analyses that were not pre-specified.

On page 8:

*“The pre-specified analysis plan is presented in S1 Text.”*

For all observational studies, in the manuscript text, please indicate: (1) the specific hypotheses you intended to test, (2) the analytical methods by which you planned to test them, (3) the analyses you actually performed, and (4) when reported analyses differ from those that were planned, transparent explanations for differences that affect the reliability of the study's results. If a reported analysis was performed based on an interesting but unanticipated pattern in the data, please be clear that the analysis was data-driven.

**Reply:** We appreciate your comment. We have already clarified our "hypotheses" in the Introduction, our "original analysis plan" in the new added S1 Text (i.e., the study protocol), our "actually performed analyses" in the revised statistical analysis section; and "any analyses differed from those that were planned" in the S1 Text.

**EDITORIAL COMMENTS**

During the internal discussion of your manuscript, we noted that the increased risk of arrhythmias appears to be attributed to undiagnosed CVD. However, there does not seem to be any evidence from the data presented that the increased risk of arrhythmias is vascular in origin, e.g. triggered by coronary artery disease due to steroid use, as implied in your cover letter. Steroids are most commonly associated with bradyarrhythmias, but in your study you report tachyarrhythmias in the study population. In addition, as you report, e.g., due to possible intestinal surgeries/stomas, disease severity seems to be as important as disease activity (i.e. outside of the acute illness). GI surgeries/stoma complications often lead to acute and chronic electrolyte disturbances, which in turn disrupt cardiac electrophysiology. In addition, many systemic treatments for IBD (other than steroids) cause renal disease of a tubular nature, which significantly alters electrolyte balance. Suggest framing your discussion/implications of your findings to reflect this including implications for long term care/follow-up/monitoring.

**Reply:** We greatly apologize for the ambiguous description in the cover letter and fully agree that further investigation on the role of IBD medications and disease activity on arrhythmias risk is important. However, the ESPRESSO cohort contains limited information on IBD medications and disease activity, therefore, unfortunately we are unable to do such analyses. We have discussed this limitation in the revised Discussion and stressed the need for further research.

On page 27:

*"Fourth, since medications (e.g., anti-TNF agents) may decrease inflammatory burden and thereby the risk of CVD (e.g., ischemic heart disease)[8], future studies are warranted to understand whether specific IBD medications could also decrease incidence of arrhythmias. However, due to limited data on IBD medications (especially biologics), potential effects of drug treatment as well as the disentanglement of drug treatment and IBD severity were not within the scope of this paper. Fifth, we lacked data on inflammatory markers, such as C-reactive protein and faecal calprotectin, to assess disease activity. "*

ABSTRACT

Abstract Background:

*Provide the context of why the study is important. The final sentence should clearly state the study question.

**Reply:** We have now modified the Background accordingly.

On page 3:

*"Although previous evidence has suggested an increased risk of cardiovascular disease (CVD) in patients with inflammatory bowel disease (IBD), its association with arrhythmias is inconclusive. In this study, we aimed to explore the long-term risk of arrhythmias in IBD patients."*

Please ensure that all numbers presented in the abstract are present and identical to numbers presented in the main manuscript text.

**Reply:** We double-checked the numbers and confirmed that they are identical.

PLOS Medicine requests that main results are quantified with 95% CIs as well as p values. Please include. When reporting p values please report as p<0.001 and where higher as the exact p value p=0.002, for example. For the purposes of transparent data reporting, if not including the aforementioned please clearly state the reasons why not.

**Reply:** Thank you for bringing this to our attention. We have added *P* values throughout the manuscript accordingly.

Please include any important dependent variables that are adjusted for in the analyses.

**Reply:** We have added the adjusted variables in the abstract.

On page 3:

*"Flexible parametric survival models estimated hazard ratios (aHR) with 95% confidence intervals (95%CIs), after adjustment for birth year, sex, county of residence, calendar year, country of birth, educational attainment, number of healthcare visits, and cardiovascular-related comorbidities."*

Throughout, suggest reporting statistical information as follows to improve clarity for the reader “22% (95% CI [13%,28%]; p</=)”. Please amend throughout the abstract and main manuscript.

**Reply:** We have modified the text accordingly.

Please note the use of commas to separate upper and lower bounds, as opposed to hyphens as these can be confused with reporting of negative values.

**Reply:** We have modified the text accordingly.

When a p value is given, please specify the statistical test used to determine it.

**Reply:** Thank you for the comment. In the Abstract, because all reported *P* values were derived from the flexible parametric survival model, we decided not to report the statistical method for each *P* value.

On page 3:

*"Flexible parametric survival models estimated hazard ratios (aHR) with 95% confidence intervals (95%CIs), after adjustment for birth year, sex, county of residence, calendar year, country of birth, educational attainment, number of healthcare visits, and cardiovascular-related comorbidities."*

Please provide brief demographic details of the study population (e.g., sex, age, ethnicity, etc.).

**Reply:** We have added the information on sex and age in the Abstract. Ethnicity, however, is unavailable in the Swedish national registers based on legal regulations.

On page 3:

*"Through a nationwide histopathology cohort, we identified patients with biopsy-confirmed IBD in Sweden during 1969-2017, including Crohn’s disease (CD: n=24,954; median age at diagnosis: 38.4 years; female: 52.2%), ulcerative colitis (UC: n=46,856; 42.1 years; 46.3%), and IBD-unclassified (IBD-U: n=12,067; 43.8 years; 49.6%), as well as their matched reference individuals and IBD-free full siblings."*

Abstract Conclusions:

*Please begin your Abstract Conclusions with "In this study, we observed ..." or similar, to summarize the main findings from your study, without overstating your conclusions. Please emphasize what is new and address the implications of your study, being careful to avoid assertions of primacy. Please avoid vague statements such as ""these results have major implications for policy/clinical care"". Mention only specific implications substantiated by the results.

**Reply:** We appreciate your suggestions, and we have revised the Abstract conclusion accordingly.

On page 4:

*"In this study, we observed that IBD patients were at an increased risk of developing arrhythmias. The excess risk persisted even 25 years after IBD diagnosis. Our findings indicate a need for awareness of this excess risk among healthcare professionals."*

AUTHOR SUMMARY

At this stage, we ask that you include a short, non-technical Author Summary of your research to make findings accessible to a wide audience that includes both scientists and non-scientists. The Author Summary should immediately follow the Abstract in your revised manuscript. This text is subject to editorial change and should be distinct from the scientific abstract. The summary should include 2-3 single sentence, individual bullet points under each of the questions.

**Reply:** We have added the Author Summary accordingly.

On page 5:

***"Author Summary***

***Why was this study done?***

- *Although previous studies have explored the associations between inflammatory bowel disease (IBD) and arrhythmias, earlier findings are inconclusive and unaddressed issues remain. For example, except for atrial fibrillation, data on the risk of other specific arrhythmias in IBD patients is lacking.*
- *Until now, the long-term risk of overall and specific arrhythmias in patients with IBD remains unclear.*

***What did the researchers find?***

- *Patients with IBD were at a higher risk of developing overall arrhythmias than the general population; the increased risk may persist even 25 years after IBD diagnosis, corresponding to one extra arrhythmia case per 80 Crohn’s disease, 58 ulcerative colitis, and 29 IBD-unclassified cases over the same period.*
- *The risks of specific arrhythmias were also increased in patients with IBD, including atrial fibrillation/flutter, other supraventricular arrhythmias, and ventricular arrhythmias/cardiac arrest.*

***What do these findings mean?***

- *Healthcare professionals should be aware of the long-term increased risk of arrhythmias in IBD patients.*
- *For those patients, a risk assessment of modifiable and established CVD risk factors could be considered."*

INTRODUCTION

l.34: Please change ‘western’ to ‘Western’.

**Reply:** Done (on page 6).

l.92: Please include a reference at the end of the statement (‘including stroke, ischemic heart disease, and venous thromboembolism event’).

**Reply:** Done (on page 6).

Please address past research and explain the need for and potential importance of your study. Indicate whether your study is novel and how you determined that. If there has been a systematic review of the evidence related to your study (or you have conducted one), please refer to and reference that review and indicate whether it supports the need for your study.

**Reply:** We appreciate your suggestion and have provided such information in the third paragraph in the Introduction (on pages 6-7).

Please conclude the Introduction with a clear description of the study question or hypothesis.

**Reply:** Thank you for the suggestion. We have stated the hypothesis in the Introduction.

On page 7:

*“Drawing from the present knowledge about the role of systemic inflammation in the development of CVD and arrhythmia [14, 15, 23], we hypothesized that patients with IBD are at an increased risk of overall arrhythmias and specific types of arrhythmias.”*

METHODS AND RESULTS

PLOS Medicine requests that main results are quantified with 95% CIs as well as p values. Please include. When reporting p values please report as p<0.001 and where higher as the exact p value p=0.002, for example. For the purposes of transparent data reporting, if not including the aforementioned please clearly state the reasons why not.

**Reply:** Thank you for bringing this to our attention. We have added *P* values accordingly.

Please include any important dependent variables that are adjusted for in the analyses.

**Reply:** Thank you for the comment. We have included the adjusted variables in the statistical analysis section.

On page 11:

*"In the population matched cohort, we conditioned the analyses on the matching variables (birth year, sex, county of residence, and calendar year) in model 1, and additionally adjusted for country of birth, educational attainment, number of healthcare visits, ischemic heart disease, heart failure, stroke, hypertension, diabetes, obesity, dyslipidemia, chronic kidney disease, and COPD in model 2."*

Suggest reporting statistical information as detailed above – see under ABSTRACT

**Reply:** We have modified the whole text accordingly.

Please present numerators and denominators for percentages, at least in the Tables [not necessarily each time they're mentioned].

**Reply:** We appreciate your comment. Although denominators for percentages were not provided in Table 2 for example, readers can calculate it through dividing "No. of events" with "percentage". Given the tables in our manuscript are already large and informative, we decided not to report denominators.

Please provide brief demographic details of the study population (e.g., sex, age, ethnicity, etc.).

**Reply:** We have added the information on sex and age in the Results. Ethnicity, however, is unavailable in the Swedish national registers due to legal regulations.

On page 13:

*"We identified 24,954 CD cases (median age at index date: 38.4 years; female: 52.2%), 46,856 UC cases (42.1 years; 46.3%), and 12,067 IBD-U cases (43.8 years; 49.6%), together with their matched reference individuals (****Table 1****)."*

l.154: Please change ‘or December 31, 2019’ to ‘or 31 December 2019’.

**Reply:** Done (on page 9).

l.158: Please define ‘PPV’.

**Reply:** Done.

On page 8:

*"Such definition yields a high positive predictive value (PPV) of 95% for IBD diagnosis [26, 27]."*

l.167: Please write ‘y’ out in full (year/s) or introduce the abbreviation at first use.

**Reply:** Done.

On pages 9-10:

*"We retrieved information on country of birth (available from 1947 onward, Nordic (including Sweden, Denmark, Finland, Norway, and Iceland) or others) from the Total Population Register [30] and educational attainment (available from 1990 onward; 4 groups:* ***0-9 years, 10-12 years, ≥13 years****, and “missing”, a proxy for socioeconomic status) from the Swedish Longitudinal Integrated Database for Health Insurance and Labour Market Studies [35]."*

ll.173-174: Please change ‘Data on cardiovascular related comorbidities preceding index date was also collected from the NPR, […]’ to ‘Data on cardiovascular-related comorbidities preceding the index date were also collected from the NPR, […]’.

**Reply:** Done (on page 10).

ll.196-197: For clarity, please change ‘[…] (<18, 18-<40, 40-<60, and ≥60), […]’ to ‘[…] (<18, 18-39, 40-59, and ≥60), […]’.

**Reply:** Done.

On page 11:

*"We calculated the risk of overall arrhythmia by sex, age at index date (<18,* ***18-39, 40-59****, and ≥60 years), calendar period at index date (1969-1989, 1990-1999, 2000-2009, and 2010-2019), educational attainment (0-9 years, 10-12 years, ≥13 years, and “missing”), and number of healthcare visits (0, 1, 2-3, and ≥4)."*

l.198: Please write ‘y’ out in full (year/s) or introduce the abbreviation at first use.

**Reply:** Done.

On page 11:

*"We calculated the risk of overall arrhythmia by sex, age at index date (<18, 18-39, 40-59, and ≥60 years), calendar period at index date (1969-1989, 1990-1999, 2000-2009, and 2010-2019), educational attainment (0-9* ***years****, 10-12* ***years****, ≥13* ***years****, and “missing”), and number of healthcare visits (0, 1, 2-3, and ≥4)."*

ll.207-210 suggest: “Third, given that the Prescribed Drug Register became available in July 2005, we restricted the analysis to individuals with index date later than 1 January 2006, and constructed models that further adjusted for the above-mentioned cardiovascular-related medications.”

**Reply:** Done.

On page 12:

*"Fifth, given that the Prescribed Drug Register became available in July 2005, we restricted the analysis to individuals with index date later than 1 January 2006, and constructed models that further adjusted for the above-mentioned cardiovascular-related medications."*

l.215: Please change ‘potential influence due to confounding’ to ‘potential influence of confounding’.

**Reply:** Done (on page 12).

ll.223-225: Under the subheading ‘Ethics consideration’, please include the information on participant consent presented in lines 141-142. In this way, readers will be able to find all information on ethical requirements in one place.

**Reply:** Done.

On page 13:

*"This study was approved by the Stockholm Ethics Review Board (2014/1287-31/4 and 2018/972-32). Individual informed consent was waived as the study was register-based [29]."*

ll.228-230: “We identified 24,954 CD cases (median age at index date: 38.4 years; female: 52.2%), 46,856 UC cases (42.1 years; 46.3%), and 12,067 IBD-U cases (43.8 years; 49.6%), together with their matched reference individuals (Table 1).” Please ensure that all parentheses indicate that the number of years is the median age at the index date and the percentage is the percentage of female participants.

**Reply:** We have double-checked the values and confirmed that all of them are correctly reported.

ll.231-233: Please change to “Compared with reference individuals, patients with IBD had a higher number of healthcare visits and a higher prevalence of previous diseases, including ischemic heart disease, heart failure, stroke, hypertension, diabetes, obesity, dyslipidemia, chronic kidney disease, and COPD.” In addition, please include a reference at the end of this statement to where this information can be found in your manuscript.

**Reply:** Done.

On page 13:

*"Compared with reference individuals, patients with IBD had a higher number of healthcare visits and a higher prevalence of previous diseases, including ischemic heart disease, heart failure, stroke, hypertension, diabetes, obesity, dyslipidemia, chronic kidney disease, and COPD (Table 1)."*

ll.239-242: Please ensure that all parentheses indicate the unit and definition of the numbers presented. It is not sufficient to put all the details in one (the first) parenthesis of the sentence and leave the complete details out of the following parentheses. Please check and revise throughout your entire manuscript.

**Reply:** We appreciate your comment. As suggested, we have revised the sentence and the entire manuscript.

On page 17:

*"During a median follow-up of around 10 years, 1,904 CD cases (7.6%; IR: 54.6 per10,000 person-years), 4,154 UC cases (8.9%; IR: 64.7 per 10,000 person-years), and 990 IBD-U cases (8.2%; IR: 78.1 per 10,000 person-years) developed overall arrhythmias, compared to 6.7% (IR: 46.1 per 10,000 person-years), 7.5% (IR: 53.3 per 10,000 person-years), and 6.0% (IR: 53.5 per 10,000 person-years) in the reference individuals (****Table 2****)."*

DISCUSSION

Please remove all subheadings from your Discussion.

**Reply:** Done.

ll.334-335: Please temper assertions of primacy ("Our study is the first one to date…”) by adding ‘to the best of our knowledge’ or similar.

**Reply:** Done.

On page 24:

*"To the best of our knowledge, our study is the first one to date to explore the long-term risk of newly diagnosed specific arrhythmias (except for atrial fibrillation) in patients with IBD."*

FIGURES

For all Figures, please ensure that you have complied with our figures requirements <http://journals.plos.org/plosmedicine/s/figures>.

**Reply:** Done.

For all Figures, including those in Supporting Information files, please show the axis beginning at zero. If this is not possible, please show a break in the axis.

**Reply:** Thank you for the comment. We have now revised all figures with the axis beginning at zero.

Please define abbreviations used in the figure legend of each figure (including those in Supporting Information files).

**Reply:** Done.

Figure 1: Please define ‘IBD’ in the figure description at first use. Also, please define ‘CD’, ‘UC’ and ‘IBD-U’ in the figure description or in a figure legend.

**Reply:** Done.

On page 20:

*"Figure 1. Hazard ratio (HR) and standardized cumulative incidence of overall arrhythmias. Left: HR and 95% confidence interval (CI) of overall arrhythmias, comparing* ***inflammatory bowel disease (IBD)*** *patients with their reference individuals; Right: standardized cumulative incidence and 95% CI of overall arrhythmias in IBD patients (pink) and their reference individuals (blue). Both HR and standardized cumulative incidence were estimated from the flexible parametric survival model.* ***CD: Crohn's disease; IBD-U: inflammatory bowel disease unclassified; UC: ulcerative colitis****."*

TABLES

Please note the use of commas to separate upper and lower bounds, as opposed to hyphens as these can be confused with reporting of negative values.

**Reply:** We appreciate your comment and have replaced all hyphen into commas.

Please define abbreviations used in the table description of each table (including those in Supporting Information files).

**Reply:** Done.

Table 1: Please define ‘y’ in the abbreviations listed below the table or change the heading to ‘Educational attainment, years’ and remove the ‘y’. Table 1: For clarity, please change the groups for ‘Age at index date’ from <18, 18-<40, 40-<60, and ≥60 to <18, 18-39, 40-59, and ≥60.

**Reply:** Done (on page 14).

Table 2: Please define ‘Pys’ in in the abbreviations listed below the table.

**Reply:** Done (on pages 19).

Table 2: Please write ‘COPD’ out in full.

**Reply:** Done (on page 19).

Supplemental Table 1: Please define ‘CVD’.

**Reply:** Done (see Table A in S1 Appendix).

Supplemental Table 2: Please define ‘SNOMED’.

**Reply:** Done (see Table B in S1 Appendix).

Supplemental Table 3: Please define ‘ICD’, ‘PSC’, ‘PI’, ‘L’ and ‘E’.

**Reply:** Done (see Table C in S1 Appendix).

Supplemental Table 3: Please remove the second comma after ‘M461,,’

**Reply:** Done (see Table C in S1 Appendix).

Supplemental Table 3: RE reference of Professor Annika Bergquist, please place the personal communication in the text. Please provide the name of the individual, the affiliation, and date of communication. The individual named must provide written permission to be named.

**Reply:** We appreciate your comment and have added a reference here to address your concern about the reference of Professor Annika Bergquist (please see it in the Table C in S1 Appendix), since this information was already published in our previous publication (**Ref 1**).

**Ref 1**: Olén O et al. Colorectal cancer in ulcerative colitis: a Scandinavian population-based cohort study. The Lancet. 2020;395(10218):123-131.

Supplemental Table 4: Please define ‘ICD’.

**Reply:** Done (see Table D in S1 Appendix).

Supplemental Table 5: Please define ‘ICD’, ‘ATC’.

**Reply:** Done (see Table E in S1 Appendix).

Supplemental Table 5: It appears you wrongfully cited Supplemental Table 4 as reference for medication ATC codes (‘Medication ATC codes: see antihypertensive medications in Supplemental Table 4’; ‘Medication ATC codes: see antidiabetic medications in Supplemental Table 4’). Instead, Supplemental Table 6 should be cited.

**Reply:** Thank you for pointing those typos out and we have now corrected them (see Table E in S1 Appendix).

Supplemental Table 7: Please define ‘COPD’.

**Reply:** Done (see Table G in S1 Appendix).

Supplemental Table 8: For clarity, please change the groups for ‘Age at index date, years’ from <18, 18-<40, 40-<60, and ≥60 to <18, 18-39, 40-59, and ≥60. Also, please define ‘y’ in the abbreviations listed below the table or change the heading to ‘Educational attainment, years’ and remove the ‘y’.

**Reply:** Done (see Table H in S1 Appendix).

Supplemental Table 8: Please define ‘Pys’ and ‘COPD’.

**Reply:** Done (see Table H in S1 Appendix).

Supplemental Table 9: Please define ‘COPD’, ‘L’ and ‘E’.

**Reply:** Done (see Table I in S1 Appendix).

Supplemental Table 10: Please define ‘COPD’, ‘L’ and ‘E’.

**Reply:** Done (see Table J in S1 Appendix).

Supplemental Table 11: Please define ‘COPD’ and remove the definition for ‘IR’ as it is not part of the table.

**Reply:** Done (see Table K in S1 Appendix).

Supplemental Table 12: Please define ‘COPD’.

**Reply:** Done (see Table L in S1 Appendix).

Supplemental Table 13: For clarity, please change the groups for ‘Age at index date, years’ from <18, 18-<40, 40-<60, and ≥60 to <18, 18-39, 40-59, and ≥60. Also, please define ‘y’ in the abbreviations listed below the table or change the heading to ‘Educational attainment, years’ and remove the ‘y’.

**Reply:** Done (see Table M in S1 Appendix).

Supplemental Table 14: Please define ‘COPD’ and ‘Pys’.

**Reply:** Done (see Table N in S1 Appendix).

REFERENCES

PLOS uses the numbered citation (citation-sequence) method and first six authors, et al. Please ensure that journal name abbreviations match those found in the National Center for Biotechnology Information (NCBI) databases (<http://www.ncbi.nlm.nih.gov/nlmcatalog/journals> ), and are appropriately formatted and capitalised. Please also see <https://journals.plos.org/plosmedicine/s/submission-guidelines#loc-references> for further details on reference formatting.

**Reply:** We appreciate your suggestion and have now formatted the references accordingly.

**Comments from the reviewers:**

**Reviewer #1:**

I would like to thank the authors for this interesting work on the risk of cardiac arrhythmias in IBD patients. The large number of patients included, the long follow-up period, and the use of a controlled matched sibling population support the results of this study despite its retrospective nature. As correctly explained in the text, the major biases include the lack of an assessment of IBD activity and the lack of details on IBD treatments, both of which have an important impact on the risk of arrhythmias. Nevertheless, the text is well written, and only minor revisions are suggested.

**Reply:** We appreciate your positive feedback.

Introduction Chapter:

-	Line 88, please replace "have" with "has".

**Reply:** Done.

-	Line 101-103. there is evidence of ventricular arrhythmias (Please add: Mubasher M et al. An Investigation into the Association Between Inflammatory Bowel Disease and Cardiac Arrhythmias: An Examination of the United States National Inpatient Sample Database. Clin Med Insights Cardiol. 2020. PMID: 33192109). Please cite it in the text with a brief description.

**Reply:** We appreciate your suggestion and have now cited and briefly descripted it in the Introduction.

On page 6:

*"First, most of the studies have primarily focused on atrial fibrillation [16-19], with one study examining ventricular arrhythmias [18]. Evidence on the risk of overall and other specific arrhythmias in IBD patients, including bradyarrhythmias, other supraventricular arrhythmias, and ventricular arrhythmias/cardiac arrest, is limited."*

-	Line 112. reference 8 "Sinh P et al. Cardiovascular Risk Assessment and Impact of Medications on Cardiovascular Disease in Inflammatory Bowel Disease" is a literature review and should not be used to confirm the lack of specific guidelines. Please remove.

**Reply:** Done.

Chapter Results:

-	The results were correctly adjusted for several variables associated with a higher risk of cardiac arrhythmias. Among them, thyroid dysfunction (both hypo- and hyperthyroidism) was not mentioned. Since the European prevalence of hypothyroidism and hyperthyroidism is about 5% (Please add: Chiovato L, et al. Hypothyroidism in Context: Where We have Been and Where We are Going. Adv Ther. 2019 PMID: 31485975) and 1% (Please add: Caputo M, et al. Incidence and prevalence of hyperthyroidism: a population-based study in the Piedmont region, Italy. Endocrine. 2020. PMID: 32056093). If available, results should also be adjusted for this parameter.

Reply: Thank you for drawing our attention to this topic. While both Grave’s and Hashimoto’s disease are immune-mediated, similarly to IBD, and the important papers cited by the reviewer above link IBD and thyroid disease, other recent data have indicated an inverse relationship with IBD (**Ref 1**). However, we agree that a sensitivity analysis adding autoimmune thyroid disease to our model is relevant. We observed a similar association between IBD and arrhythmias after further adjusting for autoimmune thyroid disease in the sensitivity analysis (see below Table 1, adapted from Table K in S1 Appendix). We have incorporated those sensitivity analyses into the Methods section, included the results in the revised Table K in S1 Appendix, and reported them in the Results section. In the revised manuscript, we have also cited the papers by Chiovato et al and Caputo et al.

Methods, on pages 11-12:

*"Fourth, since the European prevalence of hypothyroidism and hyperthyroidism is about 5% [39] and 1% [40], respectively, and both Hashimoto’s and Grave’s disease are immune-mediated (similar to IBD), we further adjusted for autoimmune thyroid disease (see* ***Table E in S1 Appendix*** *for ICD codes) in the model to assess its influence on the associations."*

Results, on page 22:

*"Robust results were observed across all sensitivity analyses after restricting the cohort to those without a diagnosis of COPD, to those without cardiovascular-related comorbidities, to those with data available on educational attainment, and to those with index date later than January 2006* ***as well as after further adjusting for autoimmune thyroid disease*** *(Table K in S1 Appendix)."*

| Table 1. Risk of arrhythmia in IBD patients and their matched population references | |
| --- | --- |
|  | HR (95%CI) |
| Outcomes | Further adjusted for autoimmune thyroid disease ^a^ |
| CD | |
| Overall arrhythmias | 1.14 (1.08, 1.21) |
| Atrial fibrillation/flutter | 1.12 (1.05, 1.19) |
| Bradyarrhythmias | 1.10 (0.94, 1.27) |
| Other supraventricular arrhythmias | 1.33 (1.13, 1.57) |
| Ventricular arrhythmias/cardiac arrest | 1.24 (1.08, 1.43) |
| UC | |
| Overall arrhythmias | 1.13 (1.09, 1.18) |
| Atrial fibrillation/flutter | 1.11 (1.07, 1.16) |
| Bradyarrhythmias | 1.03 (0.92, 1.14) |
| Other supraventricular arrhythmias | 1.31 (1.16, 1.47) |
| Ventricular arrhythmias/cardiac arrest | 1.25 (1.14, 1.37) |
| IBD-U | |
| Overall arrhythmias | 1.29 (1.19, 1.40) |
| Atrial fibrillation/flutter | 1.33 (1.21, 1.46) |
| Bradyarrhythmias | 1.21 (0.96, 1.53) |
| Other supraventricular arrhythmias | 1.36 (1.04, 1.76) |
| Ventricular arrhythmias/cardiac arrest | 1.37 (1.10, 1.70) |
| CD: Crohn's disease; CI: confidence interval; HR: hazard ratio; IBD-U: inflammatory bowel disease unclassified; UC: ulcerative colitis. | |
| ^a^ Conditioned on the matching variables (birth year, sex, county of residence, and calendar year) and further adjusted for country of birth, educational attainment, number of healthcare visits, ischemic heart disease, heart failure, stroke, hypertension, diabetes, obesity, dyslipidemia, chronic kidney disease, chronic obstructive pulmonary disease, and autoimmune thyroid disease. | |

**Ref 1**: Dore, MP et al. Clinically relevant thyroid disorders and inflammatory bowel disease are inversely related: A retrospective case-control study. Scandinavian Journal of Gastroenterology, 2021; 56(2), 171-176.

Discussion chapter:

-	Mechanism section: please write a few words about the arrhtymogenic properties of TNF, IL -1 and IL -6 (Please add: Massironi S, et al. The often overlooked cardiovascular complications of inflammatory bowel disease. Expert Rev Clin Immunol. 2023 PMID: 36722283). This is indeed an important message for future studies on the arrhythmogenic effects of specific immunosuppressants.

**Reply:** We appreciate your comments and have added this information in the revised Discussion. In the revised manuscript, we have also cited the papers by Massironi S et al.

On page 25:

*"Although the underlying mechanisms linking IBD to arrhythmias are intricate, chronic systemic inflammatory activation seems to be the key component[15]. Inflammatory cytokines, particularly tumor necrosis factor (TNF), interleukin(IL)-1, and IL-6, exert arrhythmogenic effects through directly affecting cardiac structural and electrical changes and indirectly affecting the function of other systems (e.g., liver, adipose and nervous tissue) [14,43]."*

- According to this work, there is a category of IBD patients at higher risk for cardiac arrhythmias (Chapter Discussion, Implications section", lines 402-405). Despite the lack of information on disease activity and treatment, this important message for clinicians should be presented graphically in a simple and clear picture.

**Reply:** We appreciate your suggestions. As suggested, we have added a forest plot to show the relative risk of arrhythmias in patients with IBD (for example, stratified by sex, age at index date, calendar period at index date, educational attainment, and number of healthcare visit, please see below **Figure 1**).

However, after discussion with our co-authors, at this stage, we have abstained from adding such figure to the manuscript due to three reasons: a). the relevant date were already included in the Table H in Appendix 1; b). results in Table H are more informative than such figure (for example, due to limited space, we did not show the number of events and absolute risks in the below **Figure 1**); c). using different measurement index (e.g., absolute risk or relative risk), we would identify different high-risk population for arrhythmias. For example, although the adjusted hazard ratios (i.e., relative risk) were significantly higher in UC or IBD-U patients diagnosed at 18-39 years, compared to other age groups, incidence rate (i.e., absolute risk) and its difference was largest among those diagnosed ≥60 years. Therefore, we decided not present a graph here to show the high-risk population for developing arrhythmias. However, we would certainly be willing to reconsider our position if the editors disagree with our strategy.

On page 27:

*"First, clinicians should be aware of the long-term increased risk of arrhythmias in IBD patients, such as patients with other extraintestinal manifestations and those diagnosed ≥60 years (in terms of absolute risk), which probably help recognize high-risk individual for arrhythmias at relatively early stage."*

**Figure 1**: Subgroup analysis of incident overall arrhythmias in patients with inflammatory bowel disease and their matched population references. Estimated by the flexible parametric survival model that conditioned on matching set (birth year, gender, county of residence, and calendar period) and adjusted for country of birth, educational attainment, number of healthcare visits, ischemic heart disease, heart failure, stroke, hypertension, diabetes, obesity, dyslipidemia, chronic kidney disease, and chronic obstructive pulmonary disease. CD: Crohn's disease; CI: confidence interval; HR: hazard ratio; IBD-U: inflammatory bowel disease unclassified; UC: ulcerative colitis.

**Reviewer #2:**

I read with interest this study that confirms and extend what previously known from researches on atrial fibrillation in IBD. The study design is robust and articulated and the Authors correctly acknowledged the limitations of this kind of population studies based on administrative data (i.e. lack of detail on clinical and laboratory parameters of inflammation). It would be important, as suggested, to further expand the research to the analysis of drug prescriptions to verify the hypothesis of a deleterious effect of corticosteroids and a protective one of anti-TNF on the risk of arrythmias in IBD patients.

**Reply:** We appreciate your positive feedback and fully agree that further investigation on the role of anti-TNF and other IBD medications on arrhythmias risk here is important. Unfortunately, however, the ESPRESSO cohort contains limited information on IBD medications, we are therefore unable to do such analyses. We have discussed this limitation in the revised Discussion and stressed the need for further research.

On pages 26-27:

*"Fourth, since medications (e.g., anti-TNF agents) may decrease inflammatory burden and thereby the risk of CVD (e.g., ischemic heart disease)[8], future studies are warranted to understand whether specific IBD medications could also decrease incidence of arrhythmias. However, due to limited data on IBD medications (especially biologics), potential effects of drug treatment as well as the disentanglement of drug treatment and IBD severity were not within the scope of this paper."*

**Reviewer #3:**

This is a well-conducted population-based cohort study on the long-term risk of arrhythmias in patients with inflammatory bowel disease. The study design, datasets, statistical methods and analyses, and presentation (tables and figures) and interpretation of the results are mostly adequate and of a good standard. However, they are still a couple of issues needing attention.

**Reply:** We appreciate your positive feedback.

1) Interpretation of the results. It says in the sibling comparison section on page 21 " Consistent with the primary analysis, UC or IBD-U patients demonstrated significantly higher risk of developing overall arrhythmias [aHR=1.18 (1.09-1.28) for UC; aHR=1.19 (0.99-1.42) for IBD-U]...". However, this is incorrect. Actually the results from reference comparison (Table 2) is quite different from that from sibling comparison (sTable14). The elevated risk of arrhythmia in patients with inflammatory bowel disease as compared to the reference individuals were not generally seen in sTable14 when compared to siblings apart from UC group only (with 2 of 4 subgroups still showing non-significant results). It's intriguing that the increased risk was not seen in the sibling comparison, which probably hints that risk of arrhythmia is not associated with the IBD? If one goes into the details of "Disease history before index date" sections in both Table 2 and sTable14, one can see people with IBD have higher cardiovascular risk factors even before the index IBD, and the elevated risk is more evident in the reference comparison than in the sibling comparison. Then this raises the question: are these risk factors prior to IBD or IBD itself which actually lead to the arrhythmias? Basically the sibling results make the current argument/conclusion a bit weak.

**Reply:** We thank the reviewer for this valuable comment. As suggested, we added a sensitivity analysis by excluding individuals with cardiovascular-related comorbidities before the index date from the analysis, and observed a similar association between IBD and arrhythmias (see below **Table 2**, adapted from Table K in S1 Appendix). We have incorporated those sensitivity analyses into the revised Methods section, included the results in the revised Table K in S1 Appendix, and reported them in the revised Results section.

Methods, on page 11:

*"Second, to assess the influence of cardiovascular-related comorbidities on the associations, we excluded those with cardiovascular-related comorbidities before the index date from the analysis."*

Results, on page 22:

*"Robust results were observed across all sensitivity analyses after restricting the cohort to those without a diagnosis of COPD,* ***to those without cardiovascular-related comorbidities****, to those with data available on educational attainment, and to those with index date later than January 2006 as well as after further adjusting for autoimmune thyroid disease (Table K in S1 Appendix)."*

For the difference between findings from population comparison analysis and sibling comparison analysis, we believe that it could be partially explained by unmeasured confounding shared by siblings (e.g., genetics or early environmental factors) and decreased sample size in the sibling analysis, which yields more conservative estimates and wider *CIs*.

On page 26:

*"Similarly, the conservative estimates and wider CIs observed in the sibling comparison analysis, compared to the population comparison analysis, could also be explained by unmeasured confounding shared by siblings (e.g., genetics or early environmental factors) or statistical power issue caused by decreased sample size."*

| Table 2. Risk of arrhythmia in IBD patients and their matched population references | |
| --- | --- |
|  | HR (95%CI) |
| Outcomes | Excluding those with cardiovascular-related comorbidities ^a^ |
| CD | |
| Overall arrhythmias | 1.11 (1.04, 1.19) |
| Atrial fibrillation/flutter | 1.08 (1.00, 1.17) |
| Bradyarrhythmias | 1.00 (0.83, 1.22) |
| Other supraventricular arrhythmias | 1.29 (1.08, 1.56) |
| Ventricular arrhythmias/cardiac arrest | 1.28 (1.09, 1.52) |
| UC | |
| Overall arrhythmias | 1.15 (1.09, 1.20) |
| Atrial fibrillation/flutter | 1.12 (1.07, 1.18) |
| Bradyarrhythmias | 1.05 (0.92, 1.19) |
| Other supraventricular arrhythmias | 1.27 (1.12, 1.45) |
| Ventricular arrhythmias/cardiac arrest | 1.31 (1.17, 1.47) |
| IBD-U | |
| Overall arrhythmias | 1.37 (1.22, 1.53) |
| Atrial fibrillation/flutter | 1.42 (1.25, 1.61) |
| Bradyarrhythmias | 1.45 (1.07, 1.99) |
| Other supraventricular arrhythmias | 1.14 (0.83, 1.56) |
| Ventricular arrhythmias/cardiac arrest | 1.68 (1.26, 2.23) |
| CD: Crohn's disease; CI: confidence interval; HR: hazard ratio; IBD-U: inflammatory bowel disease unclassified; UC: ulcerative colitis. | |
| ^a^ Conditioned on the matching variables (birth year, sex, county of residence, and calendar year) and further adjusted for country of birth, educational attainment, and number of healthcare visits. Cardiovascular-related comorbidities include ischemic heart disease, heart failure, stroke, hypertension, diabetes, obesity dyslipidemia, chronic kidney disease, and chronic obstructive pulmonary disease. | |

2) It is adequate to use flexible parametric survival models in the analyses. However, it would be good to write a short paragragh to introduce/explain in a bit details on why a flexible parametric survival model was chosen and how it compares to the well-known Cox model, similarity and difference and etc. The paragraph can be added as supplementary information.

**Reply:** We appreciate your suggestion and have now added this in the S2 Appendix and cited S2 Appendix in the Methods section. For your convenience, we have copied the text from the S2 Appendix below.

Methods, on page 10:

*"To estimate the average and temporal patterns of hazard ratios (HRs) and 95% CIs for incident arrhythmias in relation to IBD, flexible parametric survival models were applied to allow the effect of IBD to vary over time (time-varying effect) rather than being constant [37] (****see S2 Appendix for additional information about flexible parametric survival model****)."*

Text in the S2 Appendix:

*"Through using restricted cubic spline functions to model the baseline cumulative hazard or some other general baseline distribution in the survival analysis model, flexible parametric survival model enable proportional hazards, proportional odds and probit models to be fitted [1]. Compared to Cox regression model, flexible parametric survival model is easy to make smooth predictions (e.g., providing smooth estimates of the hazard and survival functions for any combination of covariate values), to model complicated time-dependent effects (e.g., getting any type of time-dependent hazard ratios), to model multiple time scales (i.e., in a Cox model, we usually consider only one time scale), to investigate absolute and relative effects, and to incorporate expected mortality for the relative survival analysis [2]."*

**Reviewer #4:**

The authors take on a highly relevant subject that which has received insufficient attention in the past. They apparently had access to a large cohort that allows for advanced statistics. This paper will help better understand intrinsic cardiovascular risks in IBD.

**Reply:** We appreciate your positive feedback.

I have a few concerns:

a) although Northern Europeans have a distinct understanding of Nordic countries, this could be confusion, because S, NO, FIN and DK are not the only Nordic countries, the Baltic countries would meet that definition as well let alone Canada and Russia etc. I would define this somewhere.

**Reply:** Thank you for the comment. We have defined it in the Methods section.

On page 9:

*"We retrieved information on country of birth (available from 1947 onward, Nordic (including Sweden, Denmark, Finland, Norway, and Iceland) or others) from the Total Population Register [30]".*

b) Even within S, NO, FIN and DK, not all people share the same ethnicity. I am not talking about migrants and immigrants here but the native communities in Greenland or Sámi peoples.

**Reply:** We agree that birth country does not always reflect ethnicity. However, from a research perspective, ethnicity (and religion) must not be recorded in Swedish national registers as a consequence of events during the Second World War. For that reason, data on ethnicity are unavailable. We have mentioned its unavailability in the revised limitation of our study.

On page 27:

*"Sixth, given differences in incidence and prevalence of IBD [2,4] and arrhythmias [21] across countries, regions, and ethnicities (****ethnicity is not available in our data****), and the Swedish health care system offers universal access practically free of charge, generalization of our findings to other settings should be cautious."*

c) I am not sure I fully understand how medications that have a profound impact on CV risk were considered? For instance, 5-ASA is a compound similar to ASA which has preventive effects of MACE and VTE. Please elaborate on that.

**Reply:** We agree with the reviewer that medication use is important. However, exploring the role of medications was beyond the scope of this study (which already includes 2 tables and 14 supplementary tables), but more importantly to address this objective, an alternative study design, such as propensity score matching or emulated target trial, would be necessary. Moreover, our cohort contains little information on biologics and does not have complete data on other IBD medications. We have acknowledged this as one of limitations in the revised Discussion section.

On pages 26-27:

*"Fourth, since medications (e.g., anti-TNF agents) may decrease inflammatory burden and thereby the risk of CVD (e.g., ischemic heart disease)[8], future studies are warranted to understand whether specific IBD medications could also decrease incidence of arrhythmias. However, due to limited data on IBD medications (especially biologics), potential effects of drug treatment as well as the disentanglement of drug treatment and IBD severity were not within the scope of this paper."*

d) And yes, it is a major limitation that the confounding effect of active vs. inactive disease could not be considered. This should be made more clear in the abstract

**Reply:** We appreciate your comment and have revised the abstract accordingly.

On page 4:

*"Study limitations include lack of clinical data to define IBD activity, not considering the potential role of IBD medications and disease activity, and the potential residual confounding from unmeasured factors for arrhythmias."*

**Reviewer #5:**

I have no issues with the analytic methods of the study With aHR of 1.13, 1.14 the authors need to discuss that considering case ascertainment bias by having more health care visits and that COPD contacts is not a great proxy for all smoking and since UC and CD patients are both more highly likely to have ever been smokers perhaps this is all that is being seen..its not much of a risk.

**Reply:** Thank you for the comment. We fully agree with the reviewer that COPD is not an ideal proxy for smoking and not adjusting for smoking may result in residual confounding. However, we do not believe that smoking and ascertainment bias from healthcare visit can fully explain away our findings. We have discussed their potential influences in the revised Discussion section.

On page 26:

*"Second, residual confounding is a concern because we lacked complete information on risk and protective factors for arrhythmias, including lifestyle [47] (e.g.,* ***smoking*** *and alcohol consumption) and genetic factors [48]. Nevertheless, the main results were robust after multivariable adjustment and after applying a series of sensitivity analyses. Moreover, such concern may also be partly relieved by the similar results between the population and sibling comparisons, since these factors tend to cluster within families."*

On page 26:

*"Third, ascertainment bias is unlikely to fully explain our results, since the positive association between IBD and arrhythmias persisted even 25 years after diagnosis. Moreover, such concern might be partly alleviated by adjusting for number of healthcare visit (a proxy for regular healthcare seeking behavior) in the analyses and by the similar results obtained after excluding individuals with a healthcare visit between 2 years and 6 months before the index date."*
