# Supplementary material for: Long-term risk of arrhythmias in patients with inflammatory bowel disease: A population-based, sibling-controlled cohort study
Source: PLoS Med. 2023 Oct 19;20(10):e1004305. doi: 10.1371/journal.pmed.1004305 (PMC10621936; doi:10.1371/journal.pmed.1004305)
Supplement: Attachment — Submitted filename: R2_letter to editor and reviewers_arrhythmias_final.docx [file pmed.1004305.s006.docx]

**Requests from Editors:**

**GENERAL COMMENTS**

Thank you for considered and detailed responses to editor and reviewer comments. Please see below for further minor points that we request you respond to in full.

Please revise your reporting of statistical information throughout the entire manuscript (see below for details). It is important to use a consistent and clear format for the reader. Please check your manuscript carefully for grammar, spelling and punctuation.

Reply: We have revised the reporting of the statistical information and checked for grammar, spelling and punctuation throughout the whole text accordingly.

Please replace “IBD patients” with “patients with IBD” throughout your manuscript.

Reply: Done.

Please leave no space between the superscript (note number) in the text and the word, number or mark of punctuation it follows. Please revise throughout the entire manuscript (including the Supplementary information files).

Reply: Done.

**FINANCIAL DISCLOSURE**

The funding statement should include: specific grant numbers, initials of authors who received each award, URLs to sponsors’ websites.

Reply: Done. Now the funding statement is:

*"This work was supported by FORTE (i.e., the Swedish Research Council for Health, Working Life and Welfare (*[*https://forte.se/*](https://forte.se/)*); Grant number 2016-00424 to JFL).*

*The funder of the study had no role in study design, data collection, data analysis, data*

*interpretation, or writing of the report."*

**EDITORIAL COMMENTS**

We initially suggested framing your discussion/implications of your findings to reflect implications of your study for long-term care/follow-up/monitoring. We feel this has not been sufficiently addressed and would ask you to consider these aspects in your discussion.

Reply: We appreciated your comment and have now revised the implication accordingly.

On pages 28-29 (page number in the version with track changes):

*"First, clinicians should be aware of the long-term increased risk of arrhythmias in patients with IBD, such as those with other extraintestinal manifestations and those diagnosed ≥60 years of age (in terms of absolute risk), which may help to identify high-risk individuals for arrhythmias at relatively early stage. For those high-risk patients, a long-term follow-up and a risk assessment of modifiable and established CVD risk factors should be considered, since the influence of IBD on arrhythmias is expected to escalate with the increasing burden of CVD risk factors with age."*

**ABSTRACT**

ll.59-61: Please use a consistent format when reporting statistical information (including consecutive brackets). We suggest reporting statistical information as follows to improve clarity for the reader “22% (95% CI [13%,28%]; p</=)”. Please amend throughout the abstract and main manuscript.

Reply: We appreciate your comments and have revised the whole text accordingly.

Editorial suggestion: “Compared to reference individuals, overall arrhythmias were increased in CD [54.6 vs. 46.1 per 10,000 person-years; aHR=1.15 (95% CI [1.09, 1.21]; P<0.001]), UC [64.7 vs. 53.3 per 10,000 person-years; aHR=1.14 (95% CI [1.10, 1.18]; P<0.001), and IBD-U patients [78.1 vs. 53.5 per 10,000 person-years; aHR=1.30 (95% CI [1.20, 1.41]; P<0.001).”

Reply: Done (on page 3).

**AUTHOR SUMMARY**

Please revise second subheading to ‘What Did the Researchers Do and Find?’, and describe the methods of the study in non-technical language.

Reply: We have revised this section accordingly.

On page 5:

*"What did the researchers do and find?*

- *In this population-based, sibling-controlled cohort study, we identified patients with biopsy-confirmed IBD in Sweden during 1969-2017, including Crohn’s disease (n=24,954), ulcerative colitis (n=46,856), and IBD-unclassified (n=12,067), as well as their matched reference individuals and IBD-free full siblings.*
- *…"*

Thank you for providing the Author Summary. In the final bullet point of ‘What Do These Findings Mean?’, please describe the main limitations of the study in non-technical language.

Reply: We appreciated your comment and have now added the main limitations in the Author Summary.

Abstract, on page 6:

*"Study limitations include the absence of clinical data to define IBD activity, not considering the potential role of IBD medications and disease activity, and the potential residual confounding from unmeasured factors for arrhythmias."*

Please write ‘CVD’ in full words.

Reply: Done (on page 6).

**METHODS AND RESULTS**

Thank you for providing the pre-specified analysis plan in the Methods section and providing the plan as a supplementary file (S1 Text). Please make sure that the Methods section transparently describes when analyses were planned, and when/why any data-driven changes to analyses took place. Changes in the analysis, including those made in response to peer review comments, should be identified as such in the Methods section of the paper, with rationale.

Reply: We have clearly mentioned "when the analyses were planned" in the pre-specified analysis plan (please see S1 Text, on page 2) and the changes in the analysis (in response to peer review comments, please see S1 Text, on page 5).

We have also identified those changes in the Methods section of the paper with rationale (please see the tracked version of the manuscript, on page 12, the second and the fourth sensitivity analysis).

l.265 suggest: “About 14.6% of CD cases were colonic and 15.5% of UC cases had extensive colitis (Table 1).”

Reply: Done (on page 14).

ll.270-274: For clarity and easier comparison, we suggest reporting the percentages for the CD/UC/IBD-cases and including the actual case numbers in parenthesis (i.e. switching percentages with case numbers).

Reply: Done (on page 18).

ll.275-278: Please revise the reporting of the statistical details (see above, example: “with an average aHR of 1.15 (95% CI [1.09, 1.21], P<0.001) for CD)”). Please revise throughout the entire manuscript.

Reply: We have revised the whole text accordingly.

ll.303-308: Please report exact p-values.

Reply: Exact p-values have now been reported (on pages 21-22).

ll.311-314/ll.319-324: Please report the full statistical details. Please revise throughout the entire manuscript.

Reply: We have now reported the full statistical details accordingly (on pages 22-23).

l.314: Please revise “non-significantly increased risk of bradyarrhythmias” to “There was no evidence of an significant association between IBD and bradyarrythmias” or similar.

Reply: We revised the sentence according to your suggestion (on page 22).

ll.342-343: Please report exact p-values.

Reply: Exact p-values have already been reported (on pages 23-24).

**DISCUSSION**

When discussing other studies, you gave the percentage of male participants (e.g., l.359). In your study, you consistently reported the percentage of females. We suggest changing the information in the discussion for consistency and clarity for the reader.

Reply: We appreciated your suggestion and have now reported the percentage of female participants when discussing other studies (on pages 24-25).

ll.432-437, please change to: “Fourth, since medications (e.g., anti-TNF agents) may decrease inflammatory burden and thereby the risk of CVD (e.g., ischemic heart disease) [8], future studies are warranted to understand how specific IBD medications could influence incidence of arrhythmias. However, due to limited data on IBD medications (especially biologics), potential effects of drug treatment as well as the disentanglement of drug treatment and IBD severity were not within the scope of this paper."

Reply: We revised the sentence accordingly (on page 28).

ll.438-441, please change to: Sixth, given the differences in the incidence and prevalence of IBD [2,4] and arrhythmias [21] between countries, regions, and ethnic groups (ethnicity is not available in our data), and the Swedish health care system providing universal access practically free of charge, generalization of our findings to other settings should be cautious.

Reply: We revised the sentence accordingly (on page 28).

l.445, please change to: “those aged ≥ 60 years”.

Reply: Done (on page 28).

ll.447-450, please change to: First, clinicians should be aware of the long-term increased risk of arrhythmias in IBD patients, such as those with other extraintestinal manifestations and those diagnosed at ≥60 years of age (in terms of absolute risk), which may help to identify high-risk individuals for arrhythmias at a relatively early stage.

Reply: We revised the sentence accordingly (on page 28).

ll.456-458, please change to: In addition, there are currently no specific guidelines for the assessment and management of CVD in IBD patients [8], and the robust findings of the present study underscore the urgency of developing relevant clinical guidelines.

Reply: We revised the sentence accordingly (on page 29).

ll.461-462: “Given the rise in IBD prevalence globally [4] (~0.8% Swedish population with IBD in 2017) [6,7], […]” – It appears reference 6 and 7 should be included in the parenthesis, please revise.

Reply: Done (on page 29).

**REFERENCES**

Please revise your references thoroughly. For example, in the first reference “The New England journal of medicine” should be abbreviated as “N Engl J Med”. PLOS uses the numbered citation (citation-sequence) method and first six authors, et al. Please ensure that journal name abbreviations match those found in the National Center for Biotechnology Information (NCBI) databases (<https://www.ncbi.nlm.nih.gov/nlmcatalog/journals>), and are appropriately formatted and capitalised. Please also see <https://journals.plos.org/plosmedicine/s/submission-guidelines#loc-references> for further details on reference formatting. Where website addresses are cited, please specify the date of access.

Reply: We appreciated your suggestion and have revised the references accordingly.

**FIGURES**

Figure 1: Currently, ‘CD’, ‘UC’ and ‘IBD-U’ could be mistaken for the y-axis title. For clarity, we suggest re-arranging the graph (from 3 rows/2 columns to 2 rows/3 columns). We suggest that the disease conditions function as titles for the 3 columns of graphs and that “HR (95% CI)” and “Cumulative incidence (%)” move to the left as y-axis titles (2 rows of graphs). The “years since index date” as the x-axis title should appear under each column of graphs. Subsequently, in the figure description you should change “left” and “right” to “upper row” and “lower row” or mark the two rows of graphs as “A” and “B” and describe this in the figure description accordingly.

Reply: Done.

Figure A: Please add “HR (95% CI)” to each y-axis on the left and ensure that the y-axis title is right next to y-axis (so that ‘CD’, ‘UC’ and ‘IBD-U’ do not get mistaken for the y-axis title). The x-axis title “years since index date” should appear under each column of graphs. Please ensure consistency in the number format on the y-axis (one decimal point versus none).

Reply: Done.

Figure B: Please adjust similarly to Figure A.

Reply: Done.

**TABLES**

Table 1: We note that in parentheses you provide the percentage of, for example, under 18 years old CD cases (11.5) without clarifying the meaning of the numbers in the parentheses (i.e. a unit is missing). Please revise. We suggest changing the column header to ‘XY cases/references, n (%)’ or similar (as done in Table 2).

Reply: Done (on page 15).

Table 1: In line with Reviewer 4’s comment, we suggest adding a footnote defining ‘Nordic countries’.

Reply: Done (on page 17).

Table A: For the columns “Follow-up time” and “Mean age”, please add a unit (e.g., years). Please define ‘US’ and ‘USA’. In the column “Main findings”, please ensure to report statistical information correctly and fully (e.g., mention 95% CI). Table A: The country listed for “Population-based cohort, Choi, 2019” should be Korea (not Korean).

Reply: Done.

Table B: Please change “(i.g., “M…”)” to “(e.g., “M…”).

Reply: Done.

Table D: We suggest changing the title to “Definitions of primary and secondary outcomes according to ICD codes”.

Reply: Done.

Table E: Please change “only if patient diagnosed ≥40 year” to “only if patient diagnosed ≥40 years old". Table E: We suggest changing the title to “Definitions of comorbidities according to ICD codes”.

Reply: Done.

Table F: We suggest changing the title to “Definitions of prescription medications according to ATC codes” and changing the second column header to “Definition” in line with previous tables.

Reply: Done.

Table I/J/L: We suggest adjusting the table format avoiding unnecessary line breaks to enhance accessibility (e.g., please avoid a line break in the word “References”).

Reply: Done.

Table M: In line with Reviewer 4’s comment, we suggest adding a footnote defining ‘Nordic countries’. Table M: Please see comment Table 1 (missing details/units).

Reply: Done.

**SUPPLEMENTARY MATERIAL**

S1 Text: Please ensure that in the Statistical Analysis Plan provided all abbreviations including those in the tables are defined and introduced at first use.

Reply: Done.

S2 Appendix: In the first sentence, please change ‘model’ to ‘models’. Please also change “Compared to Cox regression model, flexible parametric survival model” to “Compared with the Cox regression model, flexible parametric survival models”.

Reply: Done.

**SOCIAL MEDIA**

To help us extend the reach of your research, please provide any Twitter handle(s) that would be appropriate to tag, including your own, your coauthors’, your institution, funder, or lab. Please respond to this email with any handles you wish to be included when we tweet this paper.

Reply: please use those Twitter account: @jiangwei_sun, @ludvigsson,

@karolinskainst, @orebrouni.

**Comments from Reviewers:**

**Reviewer #1:** The paper has substantially improved and can be accepted

Reply: We appreciate your positive feedback.

**Reviewer #3:** Thanks authors for their great effort to improve the manuscript. I am satisfied with the response and revision. No further issues needing attention.

Reply: We appreciate your positive feedback.
